# Hedgehog signaling in endocrine and folliculo-stellate cells of the adult pituitary

**DOI:** 10.1530/JOE-20-0388

**Published:** 2021-01-15

**Authors:** Dominik Simon Botermann, Nadine Brandes, Anke Frommhold, Ina Heß, Alexander Wolff, Arne Zibat, Heidi Hahn, Rolf Buslei, Anja Uhmann

**Affiliations:** 1 Institute of Human Genetics, Molecular Developmental Genetics and Tumor Genetics Group, University Medical Center, Göttingen, Germany; 2 Institute of Pathology, Sozialstiftung Bamberg, Klinikum am Bruderwald, Bamberg, Germany

**Keywords:** Hedgehog, Smoothened, Patched, pituitary, folliculo-stellate cells

## Abstract

Ubiquitous overactivation of Hedgehog signaling in adult pituitaries results in increased expression of *proopiomelanocortin* (*Pomc*), *growth hormone* (*Gh*) and *prolactin* (*Prl*), elevated adrenocorticotropic hormone (Acth) production and proliferation of Sox2^+^ cells. Moreover, ACTH, GH and PRL-expressing human pituitary adenomas strongly express the Hedgehog target *GLI1.* Accordingly, Hedgehog signaling seems to play an important role in pathology and probably also in homeostasis of the adult hypophysis. However, the specific Hedgehog-responsive pituitary cell type has not yet been identified. We here investigated the Hedgehog pathway activation status and the effects of deregulated Hedgehog signaling cell-specifically in endocrine and non-endocrine pituitary cells. We demonstrate that Hedgehog signaling is unimportant for the homeostasis of corticotrophs, whereas it is active in subpopulations of somatotrophs and folliculo-stellate cells *in vivo*. Reinforcement of Hedgehog signaling activity in folliculo-stellate cells stimulates growth hormone production/release from somatotrophs in a paracrine manner, which most likely is mediated by the neuropeptide vasoactive intestinal peptide. Overall, our data show that Hedgehog signaling affects the homeostasis of pituitary hormone production via folliculo-stellate cell-mediated regulation of growth hormone production/secretion.

## Introduction

The pituitary gland is a key regulator of body homeostasis and responsible for signal exchanges between the hypothalamus and peripheral organs. Besides of the six different endocrine cell types (e.g. corticotrophs/adrenocorticotropic hormone- (Acth), somatotrophs/growth hormone- (Gh), prolactin- (Prl), thyroid-stimulating hormone-, luteinizing hormone-, follicle-stimulating hormone-secreting cells), the anterior lobe (AL) of the pituitary consists of Sox2^+^ (stem) cells and a meshwork of non-endocrine Sox2^+^ folliculo-stellate cells (FSC). The latter ones are implicated in regulation and maintenance of the endocrine cells by delivering paracrine factors (reviewed in Cox *et al.* 2017).

Hedgehog (Hh) signaling plays a major role in the development of the pituitary. However, its function in homoeostasis and disease of the adult gland is far from clear. Under normal physiological conditions Hh signaling is inactive in most cells of adult tissues. Activation occurs upon binding of Hh ligands (e.g. mammalian Sonic, Indian or Desert Hh) to the receptor protein Patched1 (Ptch). This releases the inhibition of Smoothened (Smo), which results in translocation of Smo into the primary cilium and nuclear translocation of transcription factors of the Gli family to induce target gene expression (e.g. *Gli1, Gli2* or *Ptch*) (reviewed in Bangs & Anderson 2017). Inactivation or overactivation of the pathway during pituitary organogenesis can lead to agenesis of the gland (Roessler *et al.* 2003), hypopituitarism and pituitary malformations (França *et al.* 2010, Flemming *et al.* 2013) or hyperplasia of the pituitary (Treier *et al.* 2001), respectively. Several lines of evidence additionally point toward a regulative function of the pathway in stem cell maintenance and regenerative processes in the adult pituitary. Thus, our group described the enhanced proliferation of Sox2^+^ cells in the AL after ubiquitous Hh signaling activation (Pyczek *et al.* 2016). Furthermore, other groups demonstrated that stem cells of the pituitary side population express the Hh signaling regulators Ptch and Smo (Chen *et al.* 2009, Vankelecom 2010) and that regenerative processes induce the expression of the Hh signaling target genes *Gli1* and *Gli2* in these cells (Gremeaux *et al.* 2012, Willems *et al.* 2016). Additionally, a regulatory function of Hh signaling in hormone-producing cells (e.g. corticotrophs) was proposed. Thus, Hh signaling regulates Acth expression in AtT-20 cells (Vila *et al.* 2005*a*,*b*, Pyczek *et al.* 2016) and *ex vivo* activation of the pathway in the whole pituitary leads to elevated Acth, Gh and Prl expression (Pyczek *et al.* 2016).

Additionally, there is evidence that Hh signaling is involved in hormone secretion or formation of pituitary tumors. For example patients and mice with heterozygous *PTCH/Ptch* germline mutation occasionally develop acromegaly-like symptoms (Kahn & Gordon 1967, Codish *et al.* 1973, Marcos *et al.* 1982, Cramer & Niederdellmann 1983, Bale *et al.* 1991, 1994, Kimonis *et al.* 1997, Wicking *et al.* 1997, Hahn *et al.* 1998, Lo Muzio *et al.* 1999). However, although human ACTH, GH or PRL-expressing pituitary adenoma show very high expression of the HH signaling inducer SHH and the HH target gene *GLI1* (Pyczek *et al.* 2016), a direct link between Hh signaling (e.g. mutations or pathway overactivation) and tumor formation in the AL has not been confirmed.

Altogether, our data and those from other labs strongly suggest that Hh signaling plays a role in pathology and probably in function of the adult pituitary gland, especially in corticotrophs, somatotrophs, lactotrophs and/or Sox2^+^ cells. However, it never has been analyzed whether pituitary endocrine cells and/or other cell types are Hh responders under physiological conditions. Moreover, the fact that Hh signaling is a key player in tumorigenesis and obviously also plays a role in pituitary adenoma substantiates the efforts to unravel the Hh-responsive cell type/s in the normal adult pituitary gland.

Here, we investigated the Hh signaling activation status of the adult pituitary gland on cellular level and studied the impact of a deregulated pathway in endocrine and non-endocrine pituitary cells using *in vivo* and *in vitro* approaches. By investigating mouse models for lineage tracing and for conditional cell-specific deregulation of Hh signaling we demonstrate that the Hh pathway does not play a role in corticotrophs in the adult pituitary gland. However, subpopulations of somatotrophs and FSC of the adult pituitary gland express the surrogate marker for active Hh signaling *Gli1* and descend from Gli1-expressing cells. Remarkably, we show here for the first time that activation of Hh signaling in FSC induces Gh release from somatotrophs in a paracrine manner, which most likely is mediated by the neuropeptide vasoactive intestinal peptide (Vip).

## Materials and methods

### Mice

All experiments using animals were performed in compliance with all German legal and ethical requirements and have been approved by the Lower Saxony State Office for Consumer Protection and Food Safety (file number 33.9-42502-04-15/1787). The following mouse strains were used in the study: *Ptch1^tm1Hahn^* (*Ptch^flox/flox^* (Uhmann *et al.* 2007), JAX stock # 012457), *Smo^tm2Amc^* (*Smo^flox/flox^* (Long *et al.* 2001), JAX stock # 004526), *Tg(Pomc-cre/ERT2)^#Jke^* (*PomcCreERT2* (Berglund *et al.* 2013) a kind gift from J K Elmquist), *Gli1^tm3(cre/ERT2)Alj^* (*Gli1CreERT2* (Ahn & Joyner 2004), JAX stock #007913), *Gt(ROSA)26Sor^tm9(CAG-tdTomato)Hze^* (*tdT* (Madisen *et al.* 2010), JAX stock #007905) and *Tg(S100b-EGFP)^11Lgrv^* (*S100b-EGFP* (Vives *et al.* 2003) a kind gift from C Legraverend and P Mollard).

**Figure 1:**
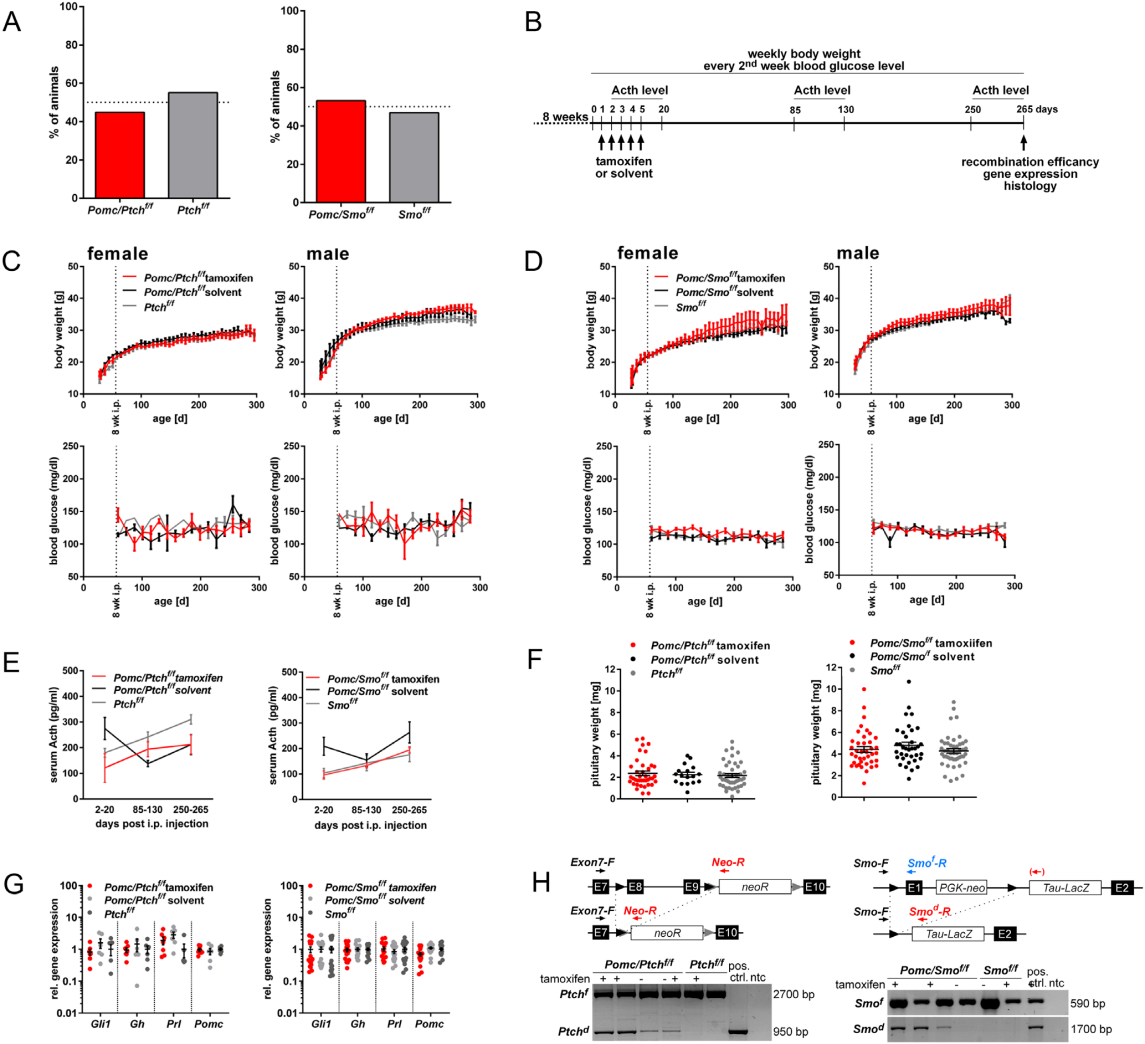
*In vivo* deregulation of Hh signaling in Pomc-expressing cells of the adult pituitary gland. (A) Percentual birth rates, (B) experimental setup, (C and D) body weight (top), blood glucose level (bottom), (E) Acth serum levels, (F) pituitary weight, (G) pituitary gene expression levels, (H) genomic loci before and after recombination (top), recombination analysis on genomic pituitary DNA (bottom) of tamoxifen- or solvent-treated *Pomc/Ptch^f/f^* and *Ptch^f/f^* or *Pomc/Smo^f/f^* and *Smo^f/f^* mice. Analyzed animal numbers are given in Supplementary Table 1, no gender-specific differences in serum Acth levels, pituitary weight or gene expression levels were observed (data not shown). Gene expression levels were normalized to *18S* rRNA expression and to the respective gene expression levels of solvent-treated controls. Each circle in F and G indicates one biological replicate (bars: mean ± s.e.m.), circles in C–E represent mean ± s.e.m. No significant differences were detected by using non-parametric Holm–Sidak method or Mann–Whitney tests.

*Ptch^flox/flox^, PomcCreERT2, tdT* and *S100b-EGFP* strains were maintained on C57BL/6 and *Smo^flox/flox^* and *Gli1CreERT2* mice on a 129/Sv background. Both genders of transgenic mice were used. No sex-specific differences were observed. Genotyping of the mice was conducted by PCR on genomic DNA isolated from tail or ear biopsies using primer pairs recommended by the donating investigators (Vives *et al.* 2003, Berglund *et al.* 2013) or by The Jackson Laboratory (https://www.jax.org/jax-mice-and-services). For CreERT2-mediated homozygous deletion of *Ptch* or *Smo Ptch^flox/flox^* or *Smo^flox/flox^* mice, respectively, were bred to the respective CreERT2-deleter mouse strain. For lineage-tracing experiments the CreERT2-deleter strains were crossed to *tdT* mice that in some experiments additionally carry the *S100b-EGFP* transgene. The CreERT2-activity of the transgenic mice was induced by five single intraperitoneal injection (i.p.) of 1 mg tamoxifen on 5 consecutive days at an animal age of 8 weeks (Uhmann *et al.* 2007). Untreated mice without the respective CreERT2-recombinase gene and solvent-treated mice carrying the floxed alleles and the respective CreERT2-recombinase genes served as controls. For lineage-tracing experiments mice were analyzed after the first tamoxifen application as indicated in the respective figure legends. Body weight and blood samples of *PomcCreERT2 Ptch^flox/flox^, PomcCreERT2 Smo^flox/flox^* and the respective control mice were taken weekly or every second week, respectively, up to 250 days after the first tamoxifen/solvent application when the mice were sacrificed (Fig. 1B). Measurements of blood glucose and serum hormone levels are described in the Supplementary methods (see section on supplementary materials given at the end of this article). The number of analyzed animals is given in Supplementary Table 1 or in the respective figure legends.

### Compounds

If not otherwise stated all compounds were obtained from Sigma-Aldrich, Darmstadt, Germany. Beta-Ala-Lys-N(epsilon)-aminomethylcoumarin acetate (β-Ala-Lys-N(ε)-AMCA) was obtained from Carbosynth (Berkshire, UK) and Smoothened Agonist (SAG) from Cayman Chemical (Ann Arbour, USA). β-Ala-Lys-N(ε)-AMCA was dissolved in HBSS (0.952 mM CaCl_2_·2H_2_O, 5.36 mM KCl, 0.411 mM KH_2_PO_4_, 0.812 mM MgSO_4_·7H_2_O, 136.7 mM NaCl, 0.385 mM Na_2_HPO_4_, 25 mM d-glucose·H_2_O, 10 mM HEPES). SAG was dissolved in dimethyl sulfoxide (DMSO). The preparation of tamoxifen/ethanol/sunflower oil for *in vivo* application has been previously described (Uhmann *et al.* 2007).

### Cell culture

GH3 (CCL-82.1, January 2016) and AtT-20 cells (CCL-89, July 2014) were obtained from ATCC and grown in Ham’s F12-K Medium (Gibco, Life Technologies) supplemented with 15% Horse serum and 2.5% heat-inactivated FBS or in Ham’s F12-K Medium (Gibco) supplemented with 15 horse serum and 2.5% FBS, respectively. TtT/GF cells were obtained from RIKEN BRC (RCB1279, September 2019) and cultured in DMEM/HamF12 (Gibco) supplemented with 10% Horse serum and 2.5% FBS. Starvation medium resembles the growth medium but contains 0.5% Horse serum and 0.125% FBS (heat inactivated for GH3 cells). Routinely, all cell lines were tested for mycoplasma contamination by using Mycoplasma Detection Kit (minerva biolabs, Berlin, Germany). Identity of the cells was analyzed by marker gene expression analyses and immunofluorescent stainings against marker proteins as shown in Supplementary Fig. 3. Passage numbers between 15 and 30 of cell lines were used for the respective experiments.

Detailed information about SAG treatment, preparation of conditioned medium, medium transfer experiments, measurements of supernatant hormone/neuropeptide levels and BrdU incorporation analysis are given in Supplementary methods.

### Detection of recombination of the *Ptch^flox^* and the *Smo^flox^* loci

Isolation of genomic DNA from pituitary glands was performed as previously described (Pyczek *et al.* 2016). For PCR-based detection of the CreERT2-mediated recombination at the *Ptch^flox^* or *Smo^flox^* locus the primer pairs indicated in Fig. 1H and  G were used. The sequences of the primers are given in Supplementary Table 2.

### RNA isolation and quantitative real-time PCR analyses

Gene expression analyses of murine tissue samples and *in vitro* cultured cells, RNA-isolation, cDNA synthesis and quantitative real-time PCR (qRT-PCR) analyses were conducted as previously described (Pyczek *et al.* 2016). All primer pairs, except those for amplification of *18S* rRNA serving for normalization of the amount of sample cDNA, were intron-flanking and are summarized in Supplementary Table 3. Each cDNA was measured in triplicates.

### Transcriptome analyses

For transcriptome analyses of three biological replicates of RNA from TtT/GF cells treated with either 100 nM SAG or solvent (see previous description) were analyzed. RNA quality control (Fragment Analyzer, Agilent Technologies), cDNA library preparation (TruSeq® RNA Sample Preparation v2; Illumina, San Diego, USA) and RNA sequencing (HiSeq 4000; Illumina) were performed at the NGS Service Facility for Integrative Genomics, Institute of Human Genetics, University Medical Center Göttingen, Germany. For detailed description see Supplementary methods. RNAseq data were deposited in the gene expression omnibus, accession: GSE153550.

### Western blot and histological analyses

Immunohistological and immunofluorescent antibody stainings of paraffin and cryosections have been described previously (Pyczek *et al.* 2016). For detailed description of protein isolation, Western blot analysis, paraffin, cryotome and vibratome sections, immunofluorescent stainings of adherent or non-adherent cells, combined RNAScope/immunofluorescent staining and β-Ala-Lys-N(ε)-AMCA incubation see Supplementary methods. Used antibodies, antibody dilutions and antigen retrieval procedures are summarized in Supplementary Table 4.

### Statistics

Statistical analyses were performed using the GraphPadPrism 6 software (GraphPad Software Inc., San Diego, USA). The used statistical tests are given in the respective figure legends.

## Results

### Deregulation of Hh signaling in **Pomc**-expressing cells has no impact on homoeostasis of adult pituitary glands

Constitutive activation of Hh signaling by a Rosa26-CreERT2-driven homozygous deletion of *Ptch* in *ex vivo* cultured adult pituitaries lead to an increase in *Pomc, Gh* and *Prl* expression and enhanced BrdU-incorporation of Sox2^+^ pituitary cells (Pyczek *et al.* 2016). Since the Rosa26-CreERT2-deleter recombines the *Ptch^flox^* locus in virtually every pituitary cell, these experiments did not allow for the determination of the specific/individual phenotype-triggering cell type. Therefore, we first tested whether Hh signaling directly regulates Acth expression in corticotrophs *in vivo*. For this purpose*,* we bred *Ptch^flox/flox^* or *Smo^flox/flox^* to *PomcCreERT2* mice, which express the tamoxifen-inducible CreERT2-recombinase under the control of the murine *proopiomelanocortin* (*Pomc*, encodes for the Acth precursor polypeptide) promoter (Berglund *et al.* 2013). To verify inducibility, specificity and potential leakiness of the deleter strain we furthermore generated *PomcCreERT2 R26-tdTomato* (*Pomc/tdT*) mice. Each mouse cohort was subdivided into two groups that received tamoxifen or solvent at an age of 8 weeks.

**Figure 2:**
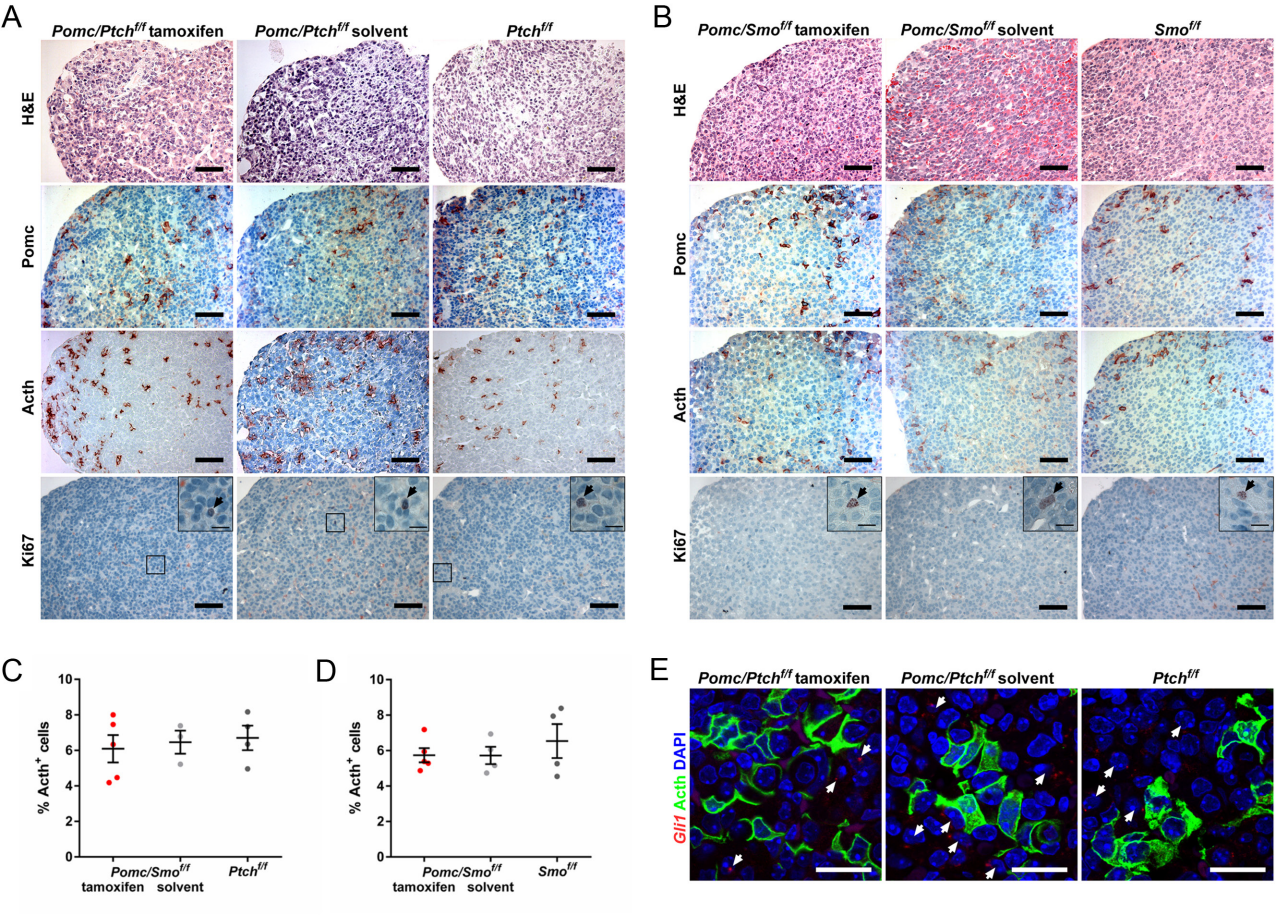
*In vivo* deregulation of Hh signaling in *Pomc*-expressing cells has no impact on the hormone expression pattern or cellular proliferation of the adult pituitary gland. (A and B) Representative (immune) histological analyses and (C and D) percentage of Acth^+^ cells of pituitaries of tamoxifen- or solvent-treated (A and C) *Pomc/Ptch^f/f^* and *Ptch^f/f^* and (B and D) *Pomc/Smo^f/f^* and *Smo^f/f^* mice 265 days post tamoxifen or solvent treatment. (E) Representative *Gli1* RNAScope/anti-Acth antibody stainings of pituitaries of tamoxifen- or solvent-treated of *Pomc/Ptch^f/f^* and *Ptch^f/f^* mice 265 days post-tamoxifen or solvent treatment (for RNAScope control stainings please see Fig. 4A). Analyzed animal numbers are given in Supplementary Table 1. Boxes: zoomed areas. Black arrows: Ki67^+^ cells. White arrows: *Gli1^+^* cells. Scale bars: 50 µm (A and B), 10 µm (A insets, B insets, E). Circles in C and D represent mean ± s.e.m. No significant differences were detected by using non-parametric Holm-Sidak method or Mann-Whitney tests.

As judged by the amount of tdT^+^ cells isolated from adult pituitaries of solvent-treated *Pomc/tdT* mice (experimental setup see Supplementary Fig. 1A), the *Pomc/tdT* reporter was highly or mildly leaky in the intermediate lobe or the AL, respectively (Supplementary Fig. 1B). However, tamoxifen-application strongly increased the number of tdT^+^ cells in the AL within 7 days after CreERT2-induction and the cells were trackable until 250 days after tamoxifen injection without any reduction of labeled cell numbers (Supplementary Fig. 1B). Double immunofluorescence analyses furthermore verified tdT expression in Pomc- and Acth- but not in Gh- or Prl-expressing cells in both tamoxifen- and solvent-treated *Pomc/tdT* mice (Supplementary Fig. 1B) indicating a cell-specific expression of the *PomcCreERT2-*transgene in corticotrophs. Thus, we expected that under normal physiological conditions the *PomcCreERT2*-deleter allows for a long-term observation of genetically modified *Pomc*-expressing cells and crossed the *PomcCreERT2-*transgene with *Ptch^flox/flox^* or *Smo^flox/flox^* mice. Irrespective of the leakiness of the deleter, *PomcCreERT2 Ptch^flox/flox^* (*Pomc/Ptch^f/f^*) and *PomcCreERT2 Smo^flox/flox^* (*Pomc/Smo^f/f^*) mice were born at a Mendelian ratio (Fig. 1A) and did not show any obvious developmental abnormities without tamoxifen application. Similarly, (experimental setup see Fig. 2B) none of the *Pomc/Ptch^f/f^* and *Pomc/Smo^f/f^* mice showed signs of a deregulated hormone status (e.g. alopecia, weight loss/gain (Fig. 1C and  D), abnormal blood glucose levels (Fig. 1C and  D), abnormal serum Acth levels (Fig. 1E) or increased pituitary weight (Fig. 1F)) 250 days after CreERT2-induction. Neither Hh signaling activity nor *Gh, Prl* or *Pomc* expression levels were altered (Fig. 1G) albeit *Ptch^flox^* or *Smo^flox^* loci were efficiently recombined in *Pomc/Ptch^f/f^* and *Pomc/Smo^f/f^* mice (Fig. 1H). In addition, no histological abnormalities were observed in *Pomc/Ptch^f/f^* and *Pomc/Smo^f/f^* pituitaries (Fig. 2A and  B, respectively). Thus, the distribution of hormone-releasing cells was normal and the *Pomc/Ptch^f/f^* or *Pomc/Smo^f/f^* pituitaries did not show any signs of hyperplasia (Fig. 2A) or hypoplasia (Fig. 2B), respectively, compared to the controls. Moreover, the percentage of Acth^+^ cells was not altered in tamoxifen-treated *Pomc/Ptch^f/f^* (Fig. 2C) or *Pomc/Smo^f/f^* mice (Fig. 2D). Finally, combined RNAScope/immunofluorescent analyses revealed that *Gli1* transcripts are not expressed in Acth-expressing cells, neither in tamoxifen-treated *Pomc/Ptch^f/f^* mice nor in the controls (Fig. 2E).

Together, *Ptch* or *Smo* depletion in *Pomc/Ptch^f/f^* and *Pomc/Smo^f/f^* pituitaries do not result in changes of Hh signaling activity or in the development of pathological phenotypes. These data show that a homozygous deletion of *Ptch* or *Smo* in *Pomc*-expressing cells has no impact on homeostasis of corticotrophs or other pituitary cells *in vivo*. These results are surprising, because *ex vivo* depletion of *Ptch* in whole pituitaries results in upregulation of *Pomc* (Pyczek *et al.* 2016).

### Somatotrophs and folliculo-stellate cells but not corticotrophs of the adult pituitary gland express *Gli1*

**Figure 3:**
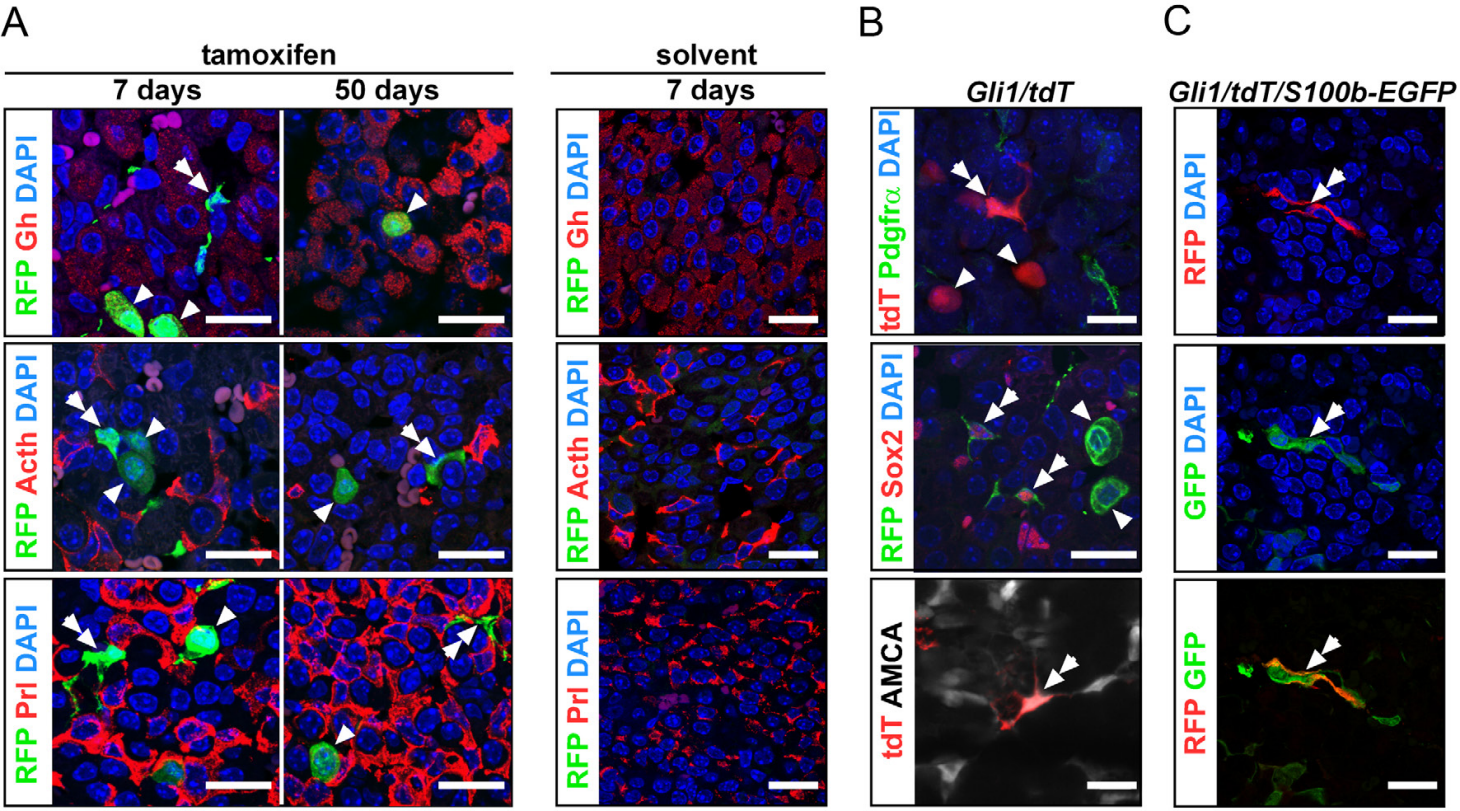
Somatotrophs and FSC of the adult pituitary gland descent from Gli1^+^ cells. Representative fluorescence analyses of adult (A and B) *Gli1/tdT* and (C) *Gli1/tdT/S100b-EGFP* pituitaries 7 or 50 days after *in vivo* tamoxifen or solvent application (A), 14 days (B bottom, C), 15 days (B top) or 22 days (B middle) days after tamoxifen injection. Similar experimental setup as shown in Fig. 1. Analyses were conducted on pituitaries of at least three animals per cohort. Arrow heads: somatotrophs; double arrows: FSC. Scale bars: 10 µm.

**Figure 4:**
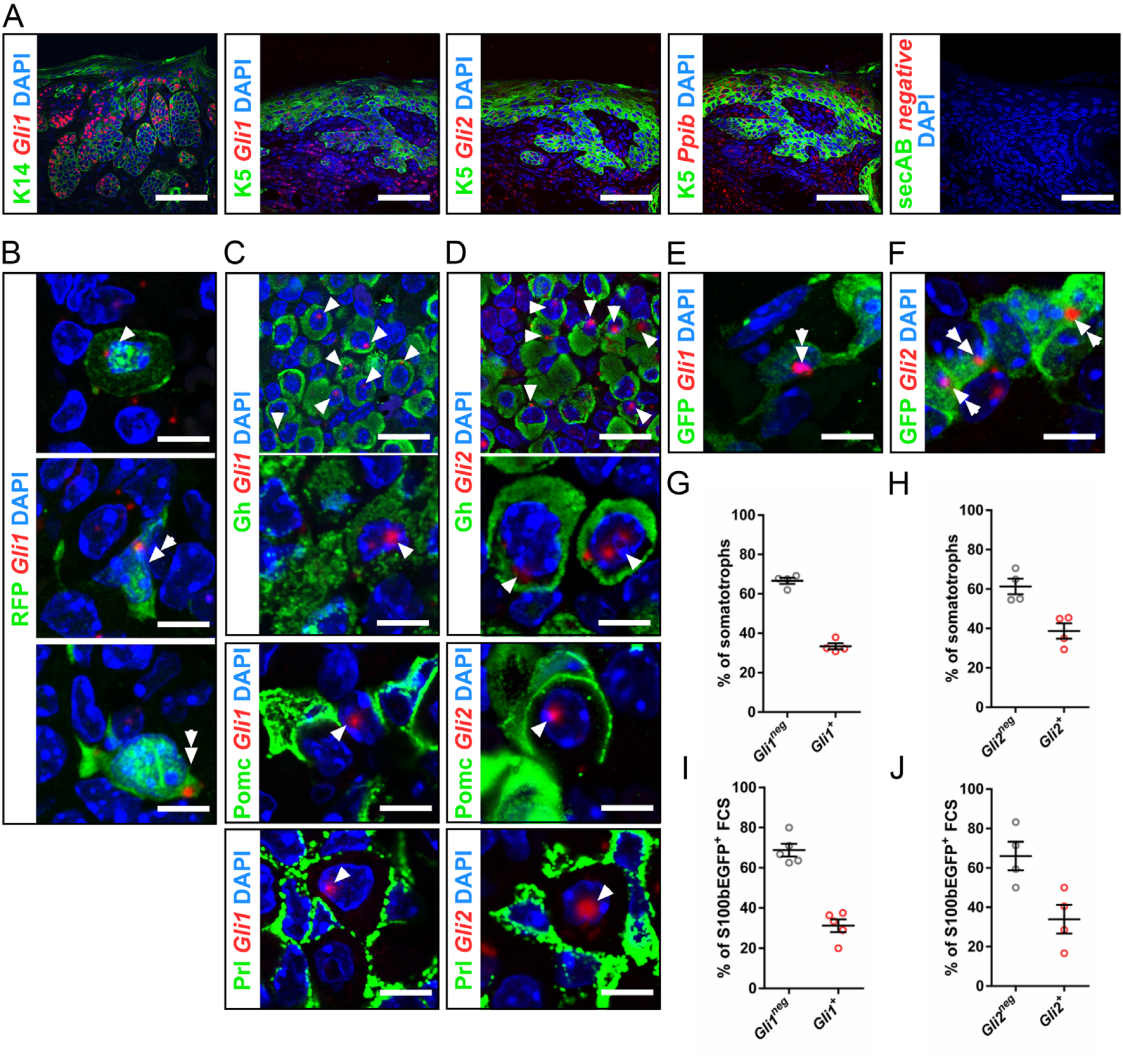
Somatotrophs and FSC of the adult pituitary gland express *Gli1* and *Gli2*. (A, B, C, D, E and F) Representative fluorescence analyses and (G, H, I and J) quantification of *Gli1^+^* (G) and *Gli2^+^* somatotrophs (H) and of *Gli1^+^* (I) and *Gli2^+^* FSC (J) of combined *Gli1* or *Gli2* transcripts detection using RNAScope technique and immunofluorescent stainings of murine basal cell carcinomas (A) (used as positive control for high *Gli1* and *Gli2* expression levels) and adult pituitary glands of tamoxifen-injected *Gli1/tdT* (B), C57BL/6N wildtype (C, D, G, H) and *S100b-EGFP* mice (E, F, I, J). Analyses were conducted on pituitaries of at least three animals per cohort. Arrow heads: somatotrops; double arrows: FSC. secAB, secondary antibody only; negative, negative control probe. Scale bars: 33 µm (A), 10 µm (C top, D top), 3.3 µm (B, C bottom, D bottom, E, F). Each open circle indicates one pituitary. Mean ± s.e.m.

Because the above-mentioned *in vivo* experiments clearly excluded a direct impact of Hh signaling at least on corticotrophs, we hypothesized that Hh signaling might regulate hormone release in an indirect manner. To shed light on this, we performed *Gli1* lineage tracing experiments by generating *Gli1CreERT2 R26-tdTomato* (*Gli1/tdT*) mice and visualized the pituitary progeny of Gli1^+^ cells under normal physiological conditions (same experimental setup as for *Pomc/tdT* mice, Supplementary Fig. 1A). Leakiness of the *Gli1CreERT2*-deleter strain was excluded by simultaneously investigated pituitary glands of solvent-treated *Gli1/tdT* mice (Fig. 3A). Remarkably, analyses of tamoxifen-treated *Gli1/tdT* adult pituitaries showed that two morphologically different pituitary cell types in the AL were marked by tdT reporter expression and thus developed from Gli1-expressing cells: one cell population with a round (Fig. 3A, arrow heads) and another with a stellate-shaped morphology (Fig. 3A, double arrows). Double immunofluorescence analyses demonstrated that the round cell type was positive for Gh and negative for Prl and Acth/Pomc, thus representing somatotrophs (Fig. 3A, arrow heads). The stellate-shaped cell type did neither express Gh, Prl, Acth (Fig. 3A) nor Pdgfra (Fig. 3B), but was positive for Sox2 (Fig. 3B) and beta-Ala-Lys-N(epsilon)-aminomethylcoumarin acetate (β-Ala-Lys-N(ε)-AMCA) (Fauquier *et al.* 2002) uptake, resembling the phenotype of FSC (Fig. 3B). To further reinforce this assumption, we additionally examined pituitaries** of tamoxifen-induced *Gli1/tdT/S100b-EGFP* mice, in which the progeny of Gli1^+^ cells and cells that express the FSC marker S100b are marked simultaneously. Indeed, this approach revealed that tdT^+^ stellate-shaped pituitary cells express EGFP (Fig. 3C) indicating that FSC represent progenies of Gli1^+^ pituitary cells. Moreover, combined RNAScope/immunofluorescent analyses (Fig. 4A, B, C, D, E and  F) and subsequent quantification of *Gli1^+^* and *Gli2^+^* cells (Fig. 4G, H, I and  J) verified that 33% (s.e.m. 1.5%) or 38% (s.e.m. 3.9%) of all somatotrophs (Fig. 4G and  H) and 31% (s.e.m. 3.2%) or 34% (s.e.m. 7.2%) of S100b-EGFP^+^ FSC (Fig. 4I and  J) express *Gli1* or *Gli2* transcripts, respectively, and thus show active Hh signaling. The distribution and number of Gli1^+^ somatotrophs and Gli1^+^ FSC and their offspring did not grossly vary between different age-matched animals.

Taken together these data demonstrate that Hh signaling is active in a constant subpopulation of somatotrophs and FSC in the adult pituitary gland and thus most likely has a function in these two pituitary cell populations.

### Hh signaling is active in the folliculo-stellate cell line TtT/GF, but not in the somatotroph cell line GH3 or in the corticotroph cell line AtT-20

Next, we studied whether the aforementioned *in vivo* data also apply to pituitary cell lines. For this purpose, we used the folliculo-stellate cell line TtT/GF, the somatotroph cell line GH3 and the corticotroph cell line AtT-20 and studied the expression of cell-specific marker genes, the basal Hh signaling activity as well as the responsiveness to Hh signaling activation. The results revealed that TtT/GF cells grow with a stellate-shaped morphology (Supplementary Fig. 2A) and express high levels of the FSC markers Sox2 (Supplementary Fig. 2A), *S100b, Vegfa, Mif* and *Fst* (Supplementary Fig. 2B), whereas GH3 or AtT-20 cells express Gh and Ghrhr (Supplementary Fig. 2C, D and E) or Pomc and Acth, respectively (Supplementary Fig. 2F and G). In contrast to GH3 and AtT-20 cells, TtT/GF cells furthermore express robust *Gli1* levels (Supplementary Fig. 3A) and show unambiguous Smo localization to primary cilia (Supplementary Fig. 3B, C and D), indicating basal Hh signaling activity. In addition, Smoothened Agonist (SAG)-treatment elevates the basal *Gli1* and *Gli2* transcription in TtT/GF (Supplementary Fig. 4A) but not in GH3 (Supplementary Fig. 4B) or AtT-20 cells (Supplementary Fig. 4C). This indicates that TtT/GF, but not GH3 or AtT-20 cells, are responsive to Hh signaling stimulation.

### Supernatant of Hh-stimulated TtT/GF folliculo-stellate cells induces Gh production in somatotroph GH3 cells, but has no impact on the corticotroph AtT-20 cell line

**Figure 5:**
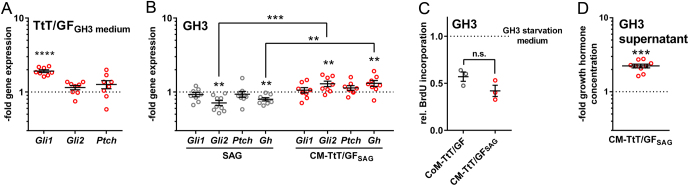
Upregulated Gh production of somatotophs via paracrine signal transduction of Hh-activated FSC. (A and B) Gene expression levels of *Gli1, Gli2, Ptch* (A and B) and *Gh* (B) of (A) TtT/GF cells after serum starvation followed by 48 h, 100 nM Smoothened Agonist (SAG) or solvent treatment (dotted line) in GH3 serum starvation medium and (B) of GH cells after serum starvation followed by 48 h, 100 nM SAG (gray circles, same data as shown in Supplementary Fig. 5B) or solvent treatment (dotted line) or by 48 h incubation with conditioned media from TtT/GF cells (shown in A) treated with SAG (CM-TtT/GF_SAG_, red circles) or solvent (dotted line). Please note: *Gli2* expression most likely is not induced by general Hh signaling activation by remaining SAG in the CM-TtT/GF_SAG_ medium since SAG-treatment of GH3 cells rather leads to reduced *Gli2* expression levels (Supplementary Fig. 5). (C) Relative BrdU incorporation of GH3 cells after serum starvation followed by 48 h incubation with GH3 starvation medium (dotted line), conditioned media from TtT/GF cells treated with SAG (CM-TtT/GF_SAG_, red circles) or solvent (CoM-TtT/GF). (D) Gh protein concentration in supernatant of GH3 cells after serum starvation followed by 48 h incubation with conditioned media from TtT/GF cells (shown in A) treated with SAG (CM-TtT/GF_SAG_, red circles) or solvent (dotted line). Gene expression levels were normalized to *18S* rRNA expression and to the respective gene expression levels of solvent-treated control cells (dotted lines). Gh concentration was normalized to the Gh concentration of solvent-treated control cells (dotted lines). Each open circle indicates one biological replicate measured in technical triplicates (A, B and D) or sextuplets (C). Mean ± s.e.m. Significant differences were tested using the non-parametric Holm-Sidak method. Significant differences to the respective base line (dotted lines) are indicated by asterisks above the data. **P = 0.01; ***P = 0.001; ****P = 0.0001; n.s., not significant.

Since FSC were responsive to Hh signaling activation, we hypothesized that active Hh signaling might indirectly influence hormone release in Gh- or Acth-expressing cells, potentially by secreted factors (reviewed in Morris & Christian 2011). To test this hypothesis, we treated GH3 or AtT-20 cells with conditioned medium from SAG-stimulated (CM-TtT/GF_SAG_) or solvent-treated TtT/GF cells (CoM-TtT/GF) (for confirmation of Hh signaling activity in TtT/GF cells after SAG treatment see Fig. 5A and Supplementary Fig. 6A), and analyzed the expression levels of *Gli1, Gli2, Ptch* and *Gh* or *Pomc*. CM-TtT/GF_SAG_-treatment neither alters Hh signaling activity in GH3 (Fig. 5B) and AtT-20 cells (Supplementary Fig. 5B), the proliferative activity of GH3 cells (Fig. 5C) nor the *Pomc* expression (Supplementary Fig. 5B) or Acth secretion level of AtT-20 cells (Supplementary Fig. 5C). However, CM-TtT/GF_SAG_-incubation significantly increases *Gh* expression levels (Fig. 5B) and Gh secretion of GH3 cells compared to the respective CoM-TtT/GF-treated controls (Fig. 5D).

These data demonstrate that Hh activation in the FSC cell line TtT/GF apparently induces the release of paracrine factors that initiate Gh production/release from GH3 cells. The factors, however, do not initiate Acth production/release from AtT-20 cells.

### Vasoactive intestinal peptide is a candidate molecule for mediating Gh production/secretion upon Hh signaling activation in folliculo-stellate cells

**Figure 6:**
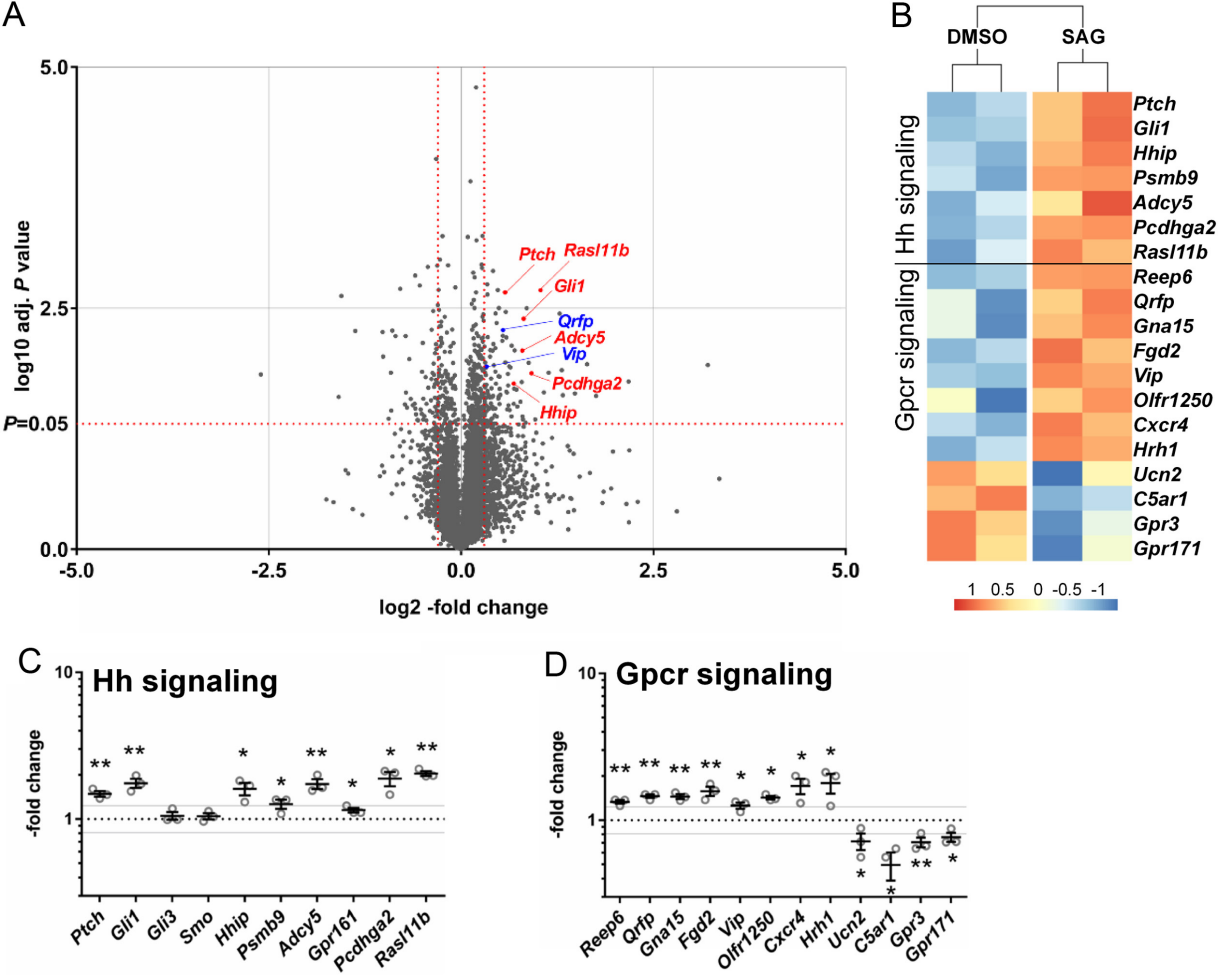
Comparative transcriptome analysis of Hh signaling activated TtT/GF cells. (A, B, C and D) Comparative transcriptome analysis of Smoothened Agonist (SAG)- vs solvent-treated (DMSO) TtT/GF cells. (A) Volcano plot of all expressed gene transcripts (cut off log^2^ -0.8- or log^2^ 1.2-fold change, red vertical lines), (B) heat map and (C and D) expression profile of significant differentially expressed genes (cut off 0.8- or 1.2-fold change, grey lines) associated with Hh and Gpcr signaling. Transcriptome analyses were conducted in biological triplicates (open circles). Gene expression of SAG-treated cells were normalized to solvent-treated controls (dotted lines in C and D). Differential expression with adjusted *P* values (non-parametric Holm–Sidak method) below 0.05 were considered to be significant (red horizontal line in A, asterisks in C and D). Significant differences to the respective baseline (dotted lines) are indicated by asterisks above/below the data. *P = 0.05; **P = 0.01; ***P = 0.001; ****P = 0.0001.

The current knowledge about the functional regulation of endocrine cells by FSC and the involved signal transduction molecules is sparse. However, growth factors and peptides may play a role in this process (Allaerts & Vankelecom 2005, Morris & Christian 2011). To identify potential candidate molecules that are upregulated upon Hh signaling activation and potentially mediate Gh production in GH3 cells in a paracrine manner, we conducted comparative transcriptome analyses of SAG- vs solvent-treated TtT/GF cells. This approach revealed that SAG-treatment leads to an up- and downregulation of 108 or 63 genes, respectively (Fig. 6A). Significantly upregulated genes included 8 genes associated with Hh signaling activation (*Ptch, Gli1, Hhip, Psmb9, Adcy5, Pcdhga2, Gpr161, Rasl11b*) (Fig. 6A, B and  C) and 8 genes associated with G protein-coupled receptors (Gpcr) signaling (*Reep6, Qrfp, Gna15, Fgd2, Vip, Olfr1250, Cxcr4, Hrh1*) (Fig. 6A, B and  D) whereas the expression of four genes associated with Gpcr signaling were downregulated (*Ucn2, C5ar1, Gpr3, Gpr171*) (Fig. 6B and  D). Additionally, SAG-treatment increased the expression levels of *insulin-like growth factor-binding protein 2* (*Igfbp2*), *angiotensin-converting enzyme* (*Ace*), *glutamate ionotropic receptor kainate type subunit 4* (*Grik4*) and the putative pituitary stem/progenitor marker* coxsackie virus and adenovirus receptor* (*Cxadr*) (Supplementary Fig. 6A). In contrast, SAG-treatment merely altered FSC marker gene expression (e.g. *Sox2, S100b, Mif, Anxa1*) (Supplementary Fig. 6B) albeit it significantly increased the transcript levels** of *Cxcr4* (Fig. 6D) and *Cxadr* (Supplementary Fig. 6A) that are also known to be expressed in FSC (Horiguchi *et al.* 2012, Chen *et al.* 2013).

**Figure 7:**
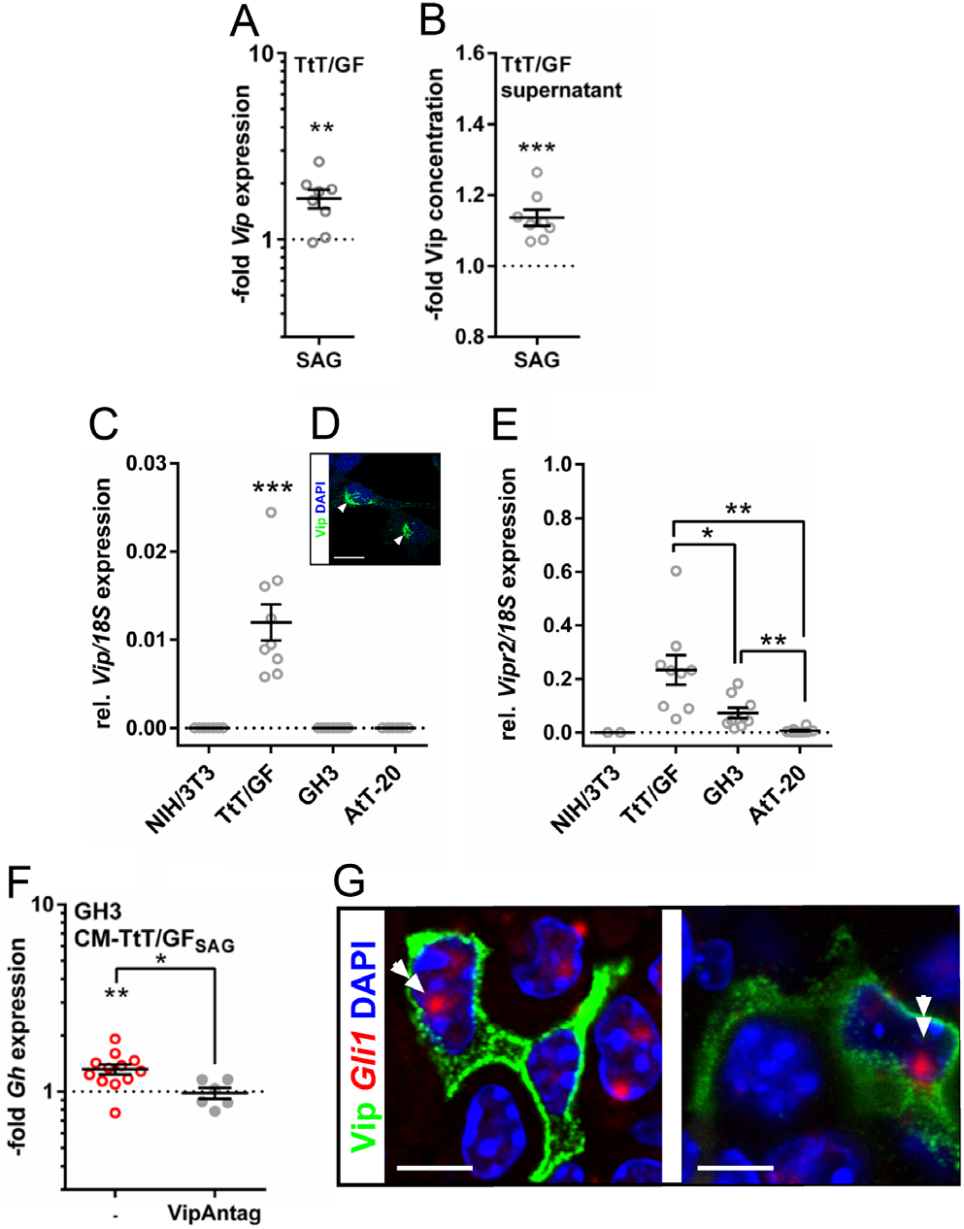
Paracrine signal transduction of Hh-activated FSC to somatotrophs is mediated by Vip/Vipr2 signaling. (A and B) qRT-PCR- (E, same samples as shown in Supplementary Fig. 5) and EIA-based (F) verification of increased *Vip*/Vip expression/secretion of SAG-treated TtT/GF cells (E) or supernatant (F, for total Vip concentration see Supplementary Fig. 8). (C, D and E) *Vip* mRNA (C), Vip protein (D) and *Vipr2* mRNA expression of TtT/GF (C, D and E), GH3 and AtT-20 cells compared to NIH/3T3 cells (C and E). (F) qRT-PCR-based analysis of *Gh* expression level of GH3 cells treated with CM-TtT/GF_SAG_ or CoM-TtT/GF supplemented with 1 µM Vip antagonist (VipAntag) or solvent, respectively. (G) Combined visualization of *Gli1* transcripts and Vip protein expression in an adult murine C57Bl6/N wildtype pituitary gland. Gene expression levels in A, C, E and F were normalized to *18S* rRNA expression and in A and F additionally to the *Vip* or *Gh* expression level of solvent-treated control cells, respectively (dotted lines in A and F). Vip protein concentration was normalized to the Vip protein concentration of solvent-treated control cells (dotted line in B). Each open circle indicates one biological replicate measured in technical triplicates. Mean ± s.e.m. Significant differences were tested using the non-parametric Holm–Sidak method. Significant differences to the respective base line (dotted lines) are indicated by asterisks above the data. *P = 0.05; **P = 0.01; ***P = 0.001. White arrows: Vip^+^ TtT/GF cells. White double arrows: *Gli1^+^* Vip^+^ stellate-shaped pituitary cells. Scale bars: 10 µm (D), 3.3 µm (G).

To this end, we focused on the two neuropeptides RF(Arg-Phe)amide family 26 amino acid peptide (Qrfp) and vasoactive intestinal peptide (Vip), which are known to regulate pituitary hormone release (Matsushita *et al.* 1981, Chihara *et al.* 1982, Abe *et al.* 1985, Denef *et al.* 1985, Bluet-Pajot *et al.* 1987, Bjoro *et al.* 1990, Alexander & Sander 1994, Mazzocchi *et al.* 1998, Vleck & Patrick 1999, Fazekas *et al.* 2000, Christian *et al.* 2007, Leprince *et al.* 2017) and whose expression levels were significantly elevated in SAG-stimulated TtT/GF cells compared to the controls (Fig. 6A, B and  D). qRT-PCR-based expression analyses verified the significant increase of *Vip* expression in SAG-treated TtT/GF cells (Fig. 7A), whereas the absolute *Qrfp* reads remained under qRT-PCR detection level. Importantly, measurement of Vip protein concentration revealed a significant increase of Vip protein in CM-TtT/GF_SAG_ compared to CoM-TtT/GF (Fig. 7B and Supplementary Fig. 7). Expression analyses of *Vip* and its receptors *Vipr1* (*vasoactive intestinal peptide receptor*), *Vipr2* and *pituitary adenylate cyclase-activating peptide* (*Pacap*) *type 1 receptor* (*Adcyap1r1*) in TtT/GF, GH3, AtT-20 and NIH/3T3 (used as negative control) cells revealed that *Vip* transcripts were only detectable in TtT/GF cells (Fig. 7C) that also showed Vip protein expression (Fig. 7D). In addition, both TtT/GF and GH3 cells showed robust *Vipr2* mRNA levels (Fig. 7E). None of the cell lines expressed *Vipr1* or *Adcyap1r1* (data not shown). Finally, we analyzed whether GH3 cells respond to Vip. Strikingly, treatment of GH3 cells with the hybrid Vip antagonist KPRRPYTDNYTRLRKQMAVKKYLNSILN-NH_2_ efficiently inhibited the CM-TtT/GF_SAG_-mediated Gh production (Fig. 7F).

These data show that Hh signaling activation in the FSC cell line TtT/GF stimulates the production and release of the neuropeptide Vip, which induces Gh production/secretion in the GH3 cells most likely via Vipr2 signaling. Moreover, the fact that *Gli1^+^* stellate-shaped pituitary cells of the adult pituitary gland also express Vip (Fig. 7G) strongly points to a similar circuit in the pituitary *in vivo*.

## Discussion

The Hh signaling pathway plays a prominent role in the development of the pituitary (Treier *et al.* 2001, Roessler *et al.* 2003, França *et al.* 2010, Flemming *et al.* 2013). However, its function in the adult gland is far from clear. Recently we demonstrated that Hh signaling activation in the adult pituitary gland leads to Acth, Gh and Prl production and proliferation of Sox2^+^ cells. Unfortunately, these experiments were not conclusive with respect to the Hh-responsive pituitary cell type in the normal gland (Pyczek *et al.* 2016). However, this information is of great importance because *GLI1* and SHH are highly expressed by GH-, PRL- and ACTH-expressing human pituitary adenoma, which suggests that HH signaling has an impact on pituitary tumor formation (Pyczek *et al.* 2016).

Here we demonstrate that a cell-specific deregulation of Hh signaling in *Pomc*-expressing cells does not affect the homeostasis of corticotrophs *in vivo*. This conclusion is based on our findings that homozygous depletion of *Ptch* or *Smo* in *Pomc*-expressing cells neither leads to defective development of the gland nor to disturbed Hh signaling activity or defective homeostasis of the adult pituitary. At the first glance, these results are contrary to our previous *ex vivo* studies on *Rosa26-CreERT2/Ptch^f/f^* pituitaries that revealed a higher Acth release upon Hh signaling activation (Pyczek *et al.* 2016). However, Rosa26-CreERT2-driven recombination targets every pituitary cell, whereas in *Pomc/Ptch^f/f^* and *Pomc/Smo^f/f^* mice Hh signaling is activated/inactivated cell-specifically in *Pomc*-expressing cells. Moreover, the fact that murine *Pomc*-expressing cells never stained positive for tdT in Gli1 lineage tracing experiments or for *Gli1* transcripts in RNAScope stainings supports the conclusion that cell-intrinsic Hh signaling is not important for corticotrophs. Currently, we cannot be completely sure whether this also applies to the human pituitary since some ACTH-expressing cells of the human pituitary are immunopositive for SHH (Vila *et al.* 2005*a*, Pyczek *et al.* 2016). Nevertheless, our new data demonstrate that *Pomc*/Acth production in corticotrophs must also involve an indirect (e.g. paracrine) effect of Hh signaling.

Beyond that, our RNAScope and Gli1 lineage tracing approaches revealed that subpopulations of somatotrophs and FSC show active Hh signaling *in vivo*. These findings are remarkable because they suggest that Hh signaling is important for homeostasis of both pituitary cell types. However, our analyses of the Hh signaling status and responsiveness toward SAG-treatment in well-accepted pituitary cell lines revealed that GH3 cells express extremely low *Gli1* levels, show very rarely ciliary Smo localization and are unresponsive to Hh signaling activation upon SAG-treatment. These facts impaired further *in vitro* analyses using GH3 cells to investigate the cell-intrinsic impact of Hh signaling in somatotrophs and the most elegant way to do so would be *in vivo* approaches. Unfortunately, until now no somatotroph-specific CreERT2-deleter mouse strains exist.

In addition, our data strongly suggest that Hh signaling influences the functionality of FSC, which activates hormone production in somatotrophs in a paracrine way. FSC represents a small (5–10%) non-hormone secreting cell population in the adult AL and are implicated in the regulation and maintenance of hormone-secreting cells by delivering paracrine factors (e.g. interleukin-6, vascular endothelial growth factor, annexin-1) (reviewed in Allaerts & Vankelecom 2005). However, the exact mechanisms of how FSC regulates endocrine cells are not well understood. Our *in vitro* approaches now demonstrate for the first time that activation of Hh signaling in the FSC cell line TtT/GF induces Gh production/secretion in GH3 cells via a paracrine mechanism. Since Vip expression and concentration are significantly increased in TtT/GF cells and in the respective supernatant after SAG-treatment, and since Vip antagonist treatment can block CM-TtT/GF_SAG_-induced Gh production from GH3 cells, this paracrine mechanism most likely encompass the neuropeptide Vip. In addition, this peptide is well known for its specific capacity to stimulate Gh production/secretion in GH3 and adenoma cells and in *in vivo* approaches (Matsushita *et al.* 1981, Chihara *et al.* 1982, Denef *et al.* 1985, Bluet-Pajot *et al.* 1987, Murakami *et al.* 1995, Fazekas *et al.* 2000). Apart from that Vip also induces Prl (Abe *et al.* 1985, Bjoro *et al.* 1990, Vleck & Patrick 1999, Fazekas *et al.* 2000, Christian *et al.* 2007) and Acth release (Alexander & Sander 1994, Mazzocchi *et al.* 1998) from the respective cell lines and endocrine and/or pituitary adenoma cells. In the normal pituitary gland Vip is expressed throughout the organ (Arnaout *et al.* 1986, Hsu *et al.* 1989) including in a so far unidentified pituitary cell type with FSC-like morphology (Hagen *et al.* 1986). Vip signal transmission into the target cells is mediated by binding to the G protein-coupled membrane-bound receptors Vipr1 or Vipr2 (type 2 receptors), but not via the Pacap-specific Pac1 receptor (type 1 receptor, encoded by *Adcyap1r1* gene) (reviewed in Hirabayashi *et al.* 2018). Interestingly, TtT/GF cells express neither *Pacap, Adcyap1r1* nor *Vipr1*. However, they express *Vip* and *Virp2* and the expression and concentration of Vip increases upon Hh signaling activation in TtT/GF cells. Most strikingly, GH3 cells express *Vipr2* but not *Vip, Adcyap1r1* or *Vipr1*. Thus, the increased Gh production/release of GH3 after incubation with CM-TtT/GF_SAG_ is most likely transmitted via Vip/Vipr2 signaling. Similar findings have been reported for the AtT-20 substrains AtT-20/D16-16 (Cellosaurus CVCL_GZ35) and AtT20/D16v (Cellosaurus CVCL_4W08), in which Vip-binding to the Vipr2 receptor induces Acth-release (Reisine *et al.* 1982, Aoki *et al.* 1997). Paternal AtT-20 cells (Cellosaurus CVCL_2300) used in our study do not express *Vipr2* (Fig. 7H). This may explain the unresponsiveness of AtT-20 cells toward Vip-enriched CM-TtT/GF_SAG_ in our setting.

Together, our data demonstrate for the first time that Hh signaling is involved in FSC-mediated regulation of Gh production/release at least *in vitro*. Moreover, our results strongly hint toward a similar role of Hh signaling *in vivo*. Nevertheless, additional studies are needed to show whether this concept is indeed transferrable to the *in vivo* situation and potentially also to Acth-expressing cells. For this purpose, *in vivo* depletion of *Gli1, Ptch* or *Smo* in FSC would be advantageous which is so far hampered by missing availability of an FSC-specific Cre- or CreERT2-deleter mouse strain. However, our findings could be of importance for several pituitary adenoma subtypes, in which HH signaling is activated (Pyczek *et al.* 2016). It is possible that Hh signaling activation in tumor-associated FSC, which are found in large numbers at the periphery of adenomas and other pituitary lesions (Nishioka *et al.* 1991, Voit *et al.* 1999, Horvath & Kovacs 2002, Cimpean *et al.* 2017) support hormone production from tumor cells. This opens the intriguing possibility that hormone production of tumor cells depends on Hh signaling activity in adjacent FSC, which thus might represent a target for future therapeutic intervention.

## Supplementary Material

Table S1: Animal numbers used for the experiments shown in Figure 2, 3 and S2.

Table S2: Oligonucleotide primers used for analyses of the recombination of the Ptchflox and the Smoflox locus.

Table S3: Oligonucleotide primers used for qRT-PCR analyses.

Table S4: Primary and secondary antibodies used for Western blot, immunhistochemical or immunofluorescent stainings of paraffine or cryotome-sections or of in vitro cultured cells.

Supplemental methods

Figure S1: Long-term characterization of the specificity and inducibility of the PomcCreERT2-deleter in adult pituitary glands. (A) Experimental setup and (B) representative immunofluorescence analyses of adult Pomc/tdT pituitaries 7, 14, 50, 100, 150, 200 and 250 days post-tamoxifen. Analyses were conducted on pituitaries of at least 3 animals per cohort. AL: anterior lobe; IL: intermediate lobe. Arrows: double positive cells. Scale bars: 500 µm (left panels), 10 µm (right panels).

Figure S2: Characterization of the murine FSC cell line TtT/GF, the rat somatotroph cell line GH3 and the murine corticotroph cell line AtT-20. (A,B) TtT/GF cells grow with a stellate-shaped morphology (A left), express the FSC- and stem cell marker Sox2 (A right) and show high expression of the FSC marker genes S100b, Vegfa, Mif and Fst (B). (C-E) GH3 cells express Gh (C, left) and Ghrhr protein (C right, E) as well as high levels of Gh transcripts (D). (F,G) AtT-20 cells express Pomc (F left) and Acth (F right) protein as well as high levels of Pomc transcripts (G). Gene expression levels were normalized to 18S rRNA expression and to the respective gene expression levels of NIH/3T3 cells (dotted lines in B and D). Pomc transcript levels of NIH/3T3, TtT/GF and GH3 remained below detection level. Each open circle indicates one biological replicate measured in technical triplicates. Mean +/- SEM. Significant differences were tested using the non-parametric Holm-Sidak method. Significant differences to the respective base line (dotted lines) are indicated by asterisks above the data. *, P=0.05; **, P=0.01; ***, P=0.001; ****, P=0.0001. glycosyl. Ghrhr: glycosylated Ghrhr variants (Chu et al., 2016). Scale bars: 200 µm (A left), 10 µm (A right, C, F)

Figure S3: Characterization of Hh signaling activity of the murine FSC cell line TtT/GF, the rat somatotroph cell line GH3 and the murine corticotroph cell line AtT-20. (A) Gli1 expression analysis of TtT/GF, GH3 and AtT-20 cells compared to the fibroblast cell line NIH/3T3. Gene expression levels were normalized to 18S rRNA expression and to the respective gene expression levels of NIH/3T3 cells (dotted line). Each open circle indicates one biological replicate measured in technical triplicates. Mean +/- SEM. Significant differences were tested using the non-parametric Holm-Sidak method. Significant differences to the respective base line (dotted lines) are indicated by asterisks above the data. *, P=0.05; ***, P=0.001; ****, P=0.0001. (B-D). Representative double immunofluorescent stainings of TtT/GF (B), GH3 (C) and AtT-20 cells (D) for analysis of the Smo location within primary cilia. Scale bars: 33 µm (B), 5 µm (C, D).

Figure S4: Smoothened Agonist treatment of the murine FSC cell line TtT/GF, the rat somatotroph cell line GH3 and the murine corticotroph cell line AtT-20. (A-C) Gene expression analyses of TtT/GF (A), GH3 (B) and AtT-20 (C) cells after serum starvation followed by 48 h 100 nM Smoothened Agonist or solvent treatment (dotted lines) dissolved in the respective starvation conditions. Gene expression levels were normalized to 18S rRNA expression and to the respective gene expression levels of solvent-treated control cells (dotted line). Each open circle indicates one biological replicate measured in technical triplicates. Mean +/- SEM. Significant differences were tested using the non-parametric Holm-Sidak method. Significant differences to the respective base line (dotted lines) are indicated by asterisks above the data. *, P=0.05; **, P=0.01; ****, P=0.0001.

Figure S5: Medium of Smoothened Agonist-stimulated TtT/GF cells does not impact on Hh signaling activity or Pomc expression levels of AtT-20 cells. Gene expression analyses of Hh signaling target genes (A,B) and Pomc (B) of (A) TtT/GF cells after serum starvation followed by 48 h 100 nM Smoothened Agonist (SAG) or solvent treatment (dotted line) in AtT-20 serum starvation medium and (B) of AtT-20 cells after serum starvation followed by 48 h 100 nM SAG (gray circles, same data as shown in Fig. S5C) or solvent treatment (dotted line) or by 48 h incubation with conditioned media from TtT/GF cells (shown in A) treated with SAG (CM-TtT/GFSAG, red circles) or solvent (dotted line). (C) Acth protein concentration in supernatant of AtT-20 cells after serum starvation followed by 48 h incubation with conditioned media from TtT/GF cells (shown in A) treated with SAG (CM-TtT/GFSAG, red circles) or solvent (dotted line). Gene expression levels were normalized to 18S rRNA expression and to the respective gene expression levels of solvent-treated control cells (dotted line). Acth concentration was normalized to the Acth concentration of solvent-treated control cells (dotted lines). Each open circle indicates one biological replicate measured in technical triplicates. Mean +/- SEM. Significant differences were tested using the non-parametric Holm-Sidak method. Significant differences to the respective base line (dotted lines) are indicated by asterisks above the data. *, P=0.05; **, P=0.01; ****, P=0.0001.

Figure S6: Graphical representation of gene expression levels of the murine FSC cell line TtT/GF after Smoothened Agonist treatment determined by comparative transcriptome analyses. Expression profile of (A) differential expressed and (B) FSC marker genes of Smoothened Agonist (SAG)- versus solvent-treated (DMSO) TtT/GF cells based on transcriptome analyses (see Fig. 7). Gene expression of SAG-treated cells were normalized to solvent-treated controls (dotted lines). Differential expression (cut off 0.8- or 1.2-fold change, grey lines) with adjusted P values (non-parametric Holm-Sidak method) below 0.05 were consider to be significant. *, P=0.05; **, P=0.01.

Figure S7: EIA-based Vip protein measurements. (A) Standard curve of the EIA-based measurement of Vip protein concentrations and (B) absolute and (C) relative Vip protein levels in the supernatant of SAG- (CM-TtT/GFSAG) or solvent-treated (CoM-TtT/GF) TtT/GF cells of 3 biological independent experiments each conducted in technical duplicates (biological replicate #1) or in triplicates (biological replicates #2 and #3). For calculation of relative Vip protein levels (C) Vip concentrations of CoM-TtT/GF of the respective biological replicates were set to 1. Grey dotted vertical lines indicate the range of Vip concentrations in the tested samples.

## Declaration of interest

The authors declare that there is no conflict of interest that could be perceived as prejudicing the impartiality of the research reported.

## Funding

Research was supported by the Deutsche Forschungsgemeinschaft http://dx.doi.org/10.13039/501100001659 to A U and R B (UH-221/6-1).

## Author contribution statement

D S B designed and performed research, collected and analyzed data, prepared the figures and wrote the manuscript. N B, A F and I H performed research and collected data. A W analyzed data, A Z analyzed data, H H contributed vital reagents and analytical tools and reviewed the paper. R B contributed vital reagents and analytical tools and reviewed the paper. A U designed research, collected and analyzed data, prepared the figures and wrote the manuscript. All authors reviewed the manuscript.
