# Supplementary material for: Hedgehog signaling in endocrine and folliculo-stellate cells of the adult pituitary
Source: J Endocrinol. 2021 Jan 15;248(3):303–16. doi: 10.1530/JOE-20-0388 (PMC7983331; doi:10.1530/JOE-20-0388)
Supplement: Table S1: Animal numbers used for the experiments shown in Figure 2, 3 and S2. [file supplementary_table_1.pdf]

Table S1: Animal numbers used for the experiments shown in Figure 2, 3 and S2.

			Pomc/Ptch$^{f/f}$		***Ptch***$^{f/f}$			***Pomc/Smo***$^{f/f}$		***Smo***$^{f/f}$
			tamoxifen	solvent	-			tamoxifen	solvent	-
**birth rate**	Fig. 1A left		88		108	Fig. 1A right		126		111
**body weight**	Fig. 1C top		> 4*	> 6*	> 3*	Fig. 1D top		> 9*	> 6*	> 8*
**blood glucose level**	Fig. 1C bottom		> 3*	> 3*	> 4*	Fig. 1D bottom		> 17*	> 22*	> 13*
**Acth serum level**	Fig. 1E	2-20 d	2	4	4	Fig. 1E	2-20 d	5	5	4
		85-130 d	5	3	4		85-130 d	5	5	4
		250-265 d	5	3	4		250-265 d	5	5	4
**pituitary weight**	Fig. 1F		39	17	44	Fig. 1F		39	36	47
**gene expression**	Fig. 1G	*Gli1* expression	7	6	5	Fig. 2G	*Gli1* expression	17	17	17
		Gli2 expression	7	6	6		*Gli2* expression	17	17	17
		Ptch expression	7	6	6		*Ptch* expression	17	17	17
		Pomc expression	7	6	6		*Pomc* expression	17	17	17
		Gh expression	7	6	6		*Gh* expression	17	17	17
		Prl expression	7	6	6		*Prl* expression	16	17	17
**immune-(histological stainings**	Fig. 2A		23	11	18	Fig. 2B		13	11	18
**% Acth$^+$ cells**	Fig. 2C		5	3	4	Fig. 2D		5	4	4
***Gli1*/Acth staining**	Fig. 2E		5	2	3					

*at each time point
