# Supplementary material for: Hedgehog signaling in endocrine and folliculo-stellate cells of the adult pituitary
Source: J Endocrinol. 2021 Jan 15;248(3):303–16. doi: 10.1530/JOE-20-0388 (PMC7983331; doi:10.1530/JOE-20-0388)
Supplement: Table S2: Oligonucleotide primers used for analyses of the recombination of the Ptchflox and the Smoflox locus. [file supplementary_table_2.pdf]

Table S2: Oligonucleotide primers used for analyses of the recombination of the *Ptch*^{flox} and the *Smo*^{flox} locus.

Name	sequence	amplicon size
Exon7-F	5‘-AGG AAG TAT ATG CAT TGG CAG GAG-3‘	*Ptch*^{f}: 2700 bp<br>*Ptch*^{d}: 950 bp
neoR	5’-GCA TCA GAG CAG CCG ATT GTC TG-3’	
Smo-F	5‘-GGG TTC CCA GGG TTG AAG ACA GCT TCG ATC TCC AG-3‘	*Smo*^{f}: 590 bp
Smo^{f}-R	5‘-GTA GCG CAA AGG CTC GCA GTG G-3‘	
Smo-F	5‘-GGG TTC CCA GGG TTG AAG ACA GCT TCG ATC TCC AG-3‘	*Smo*^{d}: 1700 bp
Smo^{d}-R	5‘-TGC CAG TTT GAG GGG ACG ACG ACA GTA TCG GCC TC-3‘	
