# Supplementary material for: Hedgehog signaling in endocrine and folliculo-stellate cells of the adult pituitary
Source: J Endocrinol. 2021 Jan 15;248(3):303–16. doi: 10.1530/JOE-20-0388 (PMC7983331; doi:10.1530/JOE-20-0388)
Supplement: Table S3: Oligonucleotide primers used for qRT-PCR analyses. [file supplementary_table_3.pdf]

Table S3: Oligonucleotide primers used for qRT-PCR analyses.

Name	sequence	location	amplicon size	species
18S-F	5’-CGC AAA TTA CCC ACT CCC G-3’	exon 1	81 bp	m/r
18S-R	5’-TTC CAA TTA CAG GGC CTC GAA-3’	exon 1		
Fn1-F	5’-TTC AAG TGT GAT CCC CAT GAA G-3’	exon 43	154 bp	m/r
Fn1-R	5’-CAG GTC TAC GGC AGT TGT CA-3’	exon 45		
Fst-F	5’-TGC TGC TAC TCT GCC AGT TC-3’	exon 1	130 bp	m/r
Fst-R	5’-GTG CTG CAA CAC TCT TCC TTG-3’	exon 2		
Gh-F	5’-AAG AGG GCA TCC AGG CTC T-3’	exon 4	111 bp	m/r
Gh-R	5’-CGT CGT CGC TGC GCA TGT T-3’	exon 5		
Gli1-F	5’-TAC ATG CTG GTG GTG CAC ATG-3’	exon 9	115 bp	m/r
Gli1-R	5’-ACC GAA GGT GCG TCT TGA GG-3’	exon 10		
Gli2-F	5’-GGT CAT CTA CGA GAC CAA CTG C-3’	exon 8	272 bp	m/r
Gli2-R	5’-GTG TCT TCA GGT TCT CCA GGC-3’	exon 9		
Hhip-F	5’-GGA GCC TTA CTT GGA CAT TCA CAA-3’	exon 4	143 bp	m/r
Hhip-R	5’-ACC GTT CCT GGT TGG TGG TAT AA-3’	exon 5		
Mif-F	5’-GCC AGA GGG GTT TCT GTC G-3’	exon 1	118 bp	m
Mif-R	5’-GTT CGT GCC GCT AAA AGT CA-3’	exon 2		
Pomc-F	5’-CAG ACC TCC ATA GAT GTG TGG AG-3’	exon 3	111 bp	m/r
Pomc-R	5’-GCG GAA GTG ACC CAT GAC GTA C-3’	exon 4		
Prl-F	5’-GAG AGC TGT TTG ACC GTG TGG-3’	exon 2	196 bp	m/r
Prl-R	5’-GAT GAC CTT GAC CAT AAA CTC AC-3’	exon 3		
Ptch-F	5’-TAC AGT CCG GGA CAG CAT ACC-3’	exon 5	152 bp	m/r
Ptch-R	5’-GTA CCC ATG GCC AAC TTC GGC TTT-3’	exon 6		
S100b-F	5’-AAC AAC GAG CTC TCT CAC TTC C-3’	exon 2	105 bp	m/r
S100b-R	5’-CTG GAA GTC ACA CTC CCC ATC-3’	exon 3		
Vip-F	5’-AGC AGA AAA TGG CAC ACC CTA-3’	exon 2	85 bp	m/r
Vip-R	5’-AAG TCT GCT GTA ATC GCT GGT-3’	exon 3		
Vegfa-F	5’-CCC ACG ACA GAA GGA GAG CAG AAG T-3’	exon 2	159 bp	m
Vegfa-R	5’-CAT CAG CGG CAC ACA GGA CGG-3’	exon 3		
Vipr2-F	5’-TAC AAC GAC CCC GAG GAT GA-3’	exon 4	85 bp	r
Vipr2-R	5’-TCA GAG AAA CAC TGT AGC CCA-3’	exon 5		
