# Supplementary material for: Hedgehog signaling in endocrine and folliculo-stellate cells of the adult pituitary
Source: J Endocrinol. 2021 Jan 15;248(3):303–16. doi: 10.1530/JOE-20-0388 (PMC7983331; doi:10.1530/JOE-20-0388)
Supplement: Table S4: Primary and secondary antibodies used for Western blot, immunhistochemical or immunofluorescent stainings of paraffine or cryotome-sections or of in vitro cultured cells. [file supplementary_table_4.pdf]

Table S4: Primary and secondary antibodies used for Western blot, immunhistochemical or immunofluorescent stainings of paraffine or cryotome-sections or of *in vitro* cultured cells.

**antigen**	**antibody**	**host**	**reactivity**	**clone/catalogue#**	**manufacturer**	**application**	**antigen retrieval**	**dilution**	**fluorochrome-labeled secondary antibody (dilution, manufacturer, catalogue#)**
**Acth**	rb anti-Acth	rb	rat	AFP-15610278	NHPP	DIF (P), IHC (P)	citric acid, pH6	1:1000	d anti-rb-Alexa488 (1:200; Jackson ImmunoResearch; #712-545-150)<br>d anti-rb-Cy3 (1:200; Jackson ImmunoResearch; #712-165-150)
**α tubulin**	ms anti α tubulin	ms	ms/h/r	DLN-0999	Dianova	WB	-	1:10,000	rb anti-ms-HRP (1:10,000; Jackson ImmunoResearch #15-035-003)
**acetylated tubulin**	ms anti su	ms	su	#T6793; clone 6-11B-1	Sigma-Aldrich	DIF (P), IHC (P), ICC	citric acid, pH6	1:100	d anti-ms-TRITC (1:200; Jackson ImmunoResearch; # 715-025-150)
**GFP**	gt anti GFP	gt	-	NB100-1770	Novus Biologicals	DIF (P), IHC (P),	citric acid, pH6	1:1000	bov anti-gt-Alexa488 (1:200; Jackson ImmunoResearch; #805-545-150)
**GFP**	rb anti GFP	rb	-	600-401-215S	Rockland	DIF (P), IHC (P),	citric acid, pH6	1:500	d anti-rb-Alexa488 (1:200; Jackson ImmunoResearch; #712-545-150)<br>d anti-rb-Cy3 (1:200; Jackson ImmunoResearch; #712-165-150)
**Gh**	rb anti-Gh	rb	rat	AFP-5641801	NHPP	DIF (P), IHC (P)	citric acid, pH6	1:500	d anti-rb-Alexa488 (1:200; Jackson ImmunoResearch; #712-545-150)<br>d anti-rb-Cy3 (1:200; Jackson ImmunoResearch; #712-165-150)
**Ghrhr**	rb anti Ghrhr	rb	ms/h/r	ABIN1386028	antibodies-online	ICC, WB	-	1:500 (ICC)<br>1:5,000 (WB)	d anti-rb-Cy3 (1:200; Jackson ImmunoResearch; #712-165-150)<br>g anti-rb-HRP (1:10,000; Jackson ImmunoResearch #111-035-045)
**K14**	ms anti-K14	ms	ms/h	ab7800	Abcam	IHC (P)	citric acid, pH6	1:1000	d anti-ms-TRITC (1:200; Jackson ImmunoResearch; #715-545-150)
**K5**	rb anti-K5	rb	ms/h	Poly19055	BioLegend	IHC (P)	citric acid, pH6	1:1000	d anti-rb-Alexa488 (1:200; Jackson ImmunoResearch; #711-545-152)
**Ki67**	ms anti-Ki67	ms	ms/h	B56	BD Pharmingen	IHC (P)	citric acid, pH6	1:50	gt anti-ms-Alexa488 (1:200; Invitrogen; #A11029)
**Pdgfrα**	gt anti-Pdgfra	gt	ms	AF1062	R&D Systems	IF (C)	-	1:20	bov anti-gt-Alexa488 (1:200; Jackson ImmunoResearch; #805-545-150)
**Pomc**	rb anti Pomc	rb	ms/h	NBP2-57719	Novus Biologicals	DIF (P), IHC (P)	citric acid, pH6	1:1000	d anti-rb-Alexa488 (1:200; Jackson ImmunoResearch; #712-545-150)<br>d anti-rb-Cy3 (1:200; Jackson ImmunoResearch; #712-165-150)
**Prl**	rb anti Prl	rb	ms	AFP-425-10-91	NHPP	DIF (P), IHC (P)	citric acid, pH6	1:1000	d anti-rb-Alexa488 (1:200; Jackson ImmunoResearch; #712-545-150)<br>d anti-rb-Cy3 (1:200; Jackson ImmunoResearch; #712-165-150)

**RFP***	gt anti-RFP	gt	ms	MBS448122	MyBioSource	DIF (P)	citric acid, pH6	1:200	bov anti-gt-Alexa488 (1:200; Jackson ImmunoResearch; #805-545-150)
**RFP***	rb anti-RFP	gt	ms	600-401-379S	Rockland	DIF (P)	citric acid, pH6	1:500	d anti-rb-Alexa488 (1:200; Jackson ImmunoResearch; #712-545-150)<br>d anti-rb-Cy3 (1:200; Jackson ImmunoResearch; #712-165-150)
**Smoothened**	rb anti Smo	rb	ms/rat/h	ab38686	Abcam	DIF (P), ICC	citric acid, pH6	1:1000	d anti-rb-Alexa488 (1:200; Jackson ImmunoResearch; #712-545-150)<br>d anti-rb-Cy3 (1:200; Jackson ImmunoResearch; #712-165-150)
**Sox2**	rat anti-Sox2	rat	ms/h	14-9811	eBioscience	DIF (P), ICC	citric acid, pH6	1:100	d anti-rat-Cy3 (1:200; Jackson ImmunoResearch; # 712-165-150)
**Vip**	rb anti Vip	rb	ms/r/h	ab272726	Abcam	DIF (P), ICC	citric acid, pH6	1:500	d anti-rb-Alexa488 (1:200; Jackson ImmunoResearch; #712-545-150)

* detects tdTomato; m: mouse, r: rat; rb: rabbit; g: goat; d: donkey; IF: immunofluorescence; DIF: double immunofluorescence; IHC: immunohistochemistry; WB: Western blot; P: paraffine sections; C: cryosectios
