# Supplementary material for: Hedgehog signaling in endocrine and folliculo-stellate cells of the adult pituitary
Source: J Endocrinol. 2021 Jan 15;248(3):303–16. doi: 10.1530/JOE-20-0388 (PMC7983331; doi:10.1530/JOE-20-0388)
Supplement: Supplemental methods [file supplementary_table_5.pdf]

Supplemental methods

SAG treatment, preparation of conditioned medium and medium transfer experiments, BrdU incorporation analysis

For SAG treatment, $5x10^4$ TtT/GF, $4x10^5$ GH3 or $1x10^5$ AtT-20 cells per well of 6-well-plates were seeded in the respective normal growth medium. 24 h after seeding the cells were starved for 24 h in the respective starvation medium followed by 48 h incubation in starvation medium supplemented with 100 nM SAG or solvent (DMSO). Cells and supernatant were collected for RNA isolation or neuropeptide level measurement, respectively.

For preparation of conditioned medium $5x10^4$ TtT/GF cells per well of a 6-well plate were seeded in normal growth medium. 24 h after seeding the cells were starved for 24 h and subsequently cultured for 48 h in the respective medium of the target cell line (GH3 or AtT-20 starvation medium) supplemented with 100 nM SAG (CM-TtT/GF_{SAG}) or solvent (CoM-TtT-GF). Afterwards the cells were harvested for RNA isolation and the supernatant was filtered through a 0.22 µm filter and stored at 4 °C until further use (maximum 14 days). For expression and supernatant analysis $4x10^5$ GH3 and $1x10^5$ AtT-20 cells per well of a 6-well-plate and for BrdU incorporation 5,000 GH3 cells per well of a 96-well-plate were seeded in their respective normal growth medium. 24 h after seeding the growth medium was discarded, cells were starved for 24 h in their respective starvation medium followed by incubation with 1 ml or 100 µl CM-TtT/GF_{SAG} or CoM-TtT-GF per well of a 6-well-plate or a 96-well-plate, respectively, for 48 h. Afterwards the cells and the supernatants were collected for RNA isolation or hormone level measurement, respectively. For Vip antagonist (KPRRPYTDNYTRLRKQMAVKKYLNSILN-NH_2 , Bachem, Switzerland) treatment, GH3 cells were starved as described above and then pretreated with 1 µM Vip antagonist in GH3 starvation medium for 1h. Afterwards the cells were

incubated for 48h with CM-TtT/GF_{SAG} or CoM-TtT/GF supplemented with 1 µM Vip antagonist or the respective volume of solvent (water). BrdU pulsing (24 h prior analysis) and BrdU incorporation analysis was performed as recently described (Pyczek et al., 2016).

Paraffin, cryotome and vibratome sections

For histological analyses tissue samples were fixed in 4% paraformaldehyde, embedded in Cryo Embedding Medium (Medite Inc., Burgdorf, Germany) or dehydrated and embedded in paraffin and sectioned on a cryotome (Leica Microsysteme, Germany) or a sliding microtome (Reichart-Jung, Germany), respectively. For vibratome sections of freshly isolated murine pituitary glands, the glands were embedded in 2 % low melting agarose (Carl Roth, Karlsruhe, Germany) and placed in HBSS. 200 µm sections were made on a LEICA VT1000S vibratome (Leica, Wetzlar, Germany). The sections were directly incubated with 50 µM β-Ala-Lys-N(ε)-AMCA dissolved in HBSS at 37°C, 5 % CO_2 for 3 hrs. Afterwards fixed in 4 %paraformaldehyde for 10 min and mounted in Mowiol (4,3 mM Mowiol 4-88, 3016,24 mM Glycerol, 18502,811 mM H_2O, 133 mM Tris pH 8.5, 34,05 mM 1,4-diazabicyclo[2.2.2]octane).

Western blot analysis

Protein isolation and Western blot analysis were conducted as previously described (Becker et al., 2020).

Measurements of blood glucose, serum and supernatant hormone/neuropeptide levels

Blood glucose concentration was measured every other week between 9 am and 10 am with a blood glucose meter (Contour XT, Bayer, Leverkusen, Germany) using 10 µl blood from the murine retroorbital plexus. For Acth measurements 150 µl of blood were collected from the retroorbital plexus at specific time points during the observation period (see Fig. S2C). The blood was coagulated for two hours at 4 °C and centrifuged for 10 min at 3,000 rpm. The serum was removed and stored at -80 °C until use. Acth concentration in serum or supernatants was measured in duplicates or tripicates with an ELISA for Acth (Biomatik Corpoaration, Cambridge, Canada) according to manufacturer’s instructions using a microplate reader (BioTek, Vermont, USA). Gh and Vip levels in the supernatant of cultured cells were measured in triplicates using a Gh ELISA (Biomatik Corpoaration) or Vip EIA (Phoenix Pharmaceuthicals, Inc., Burlingame, USA) according to manufacturer’s instructions by using a microplate reader (BioTek, Vermont, USA).

RNAScope, immunohistological and immunofluorescent antibody stainings

Immunohistological and immunofluorescent antibody stainings of paraffin and cryosections have been described previously (Pyczek et al., 2016). For immunofluorescent stainings of adherent or non-adherent cells they were cultured on poly-L-lysine (Sigma-Aldrich) coated coverslips or on SHI-FIX^{TM} (Everest Biotech, Oxfordshire, UK) coverslips, respectively. Afterwards the cells were fixed with 4 %paraformaldehyde for 10 min, incubated with PBS containing 0.5 % Triton X-100 for 30 min. Blocking was performed with 0.2 % (w/v) I-Block (Applied Biosystems, Waltham, USA) in TBS for 30 min. The cells were incubated for 1 hr with the primary antibody and 45 min with the secondary antibody at RT diluted in PBS. Specimens

were mounted with ProLong™ Gold Antifade Mountant with DAPI (Invitrogen, Carlsbad, USA). Used antibodies, antibody dilutions and antigen retrieval procedures are summarized in Table S4.

For quantification of corticotrophs, $Acth^+$ cells of 1,600-3,300 nucleated cells per murine AL mouse pituitary (the numbers of analysed animals are given in Suppl. Table S1) were counted.

RNAscope® analyses of paraffin-embedded murine samples were performed using RNAscope®Probe Mm-Gli1 (311001), Mm-Gli2 (405771), Mm-Ppib (313911), Negative Control Probe-DapB (310043) and the RNAscope® 2.5 HD Reagent Kit-RED (322350; Advanced Cell Diagnostics, Newark, USA) according to the manufacturer's recommendations. In brief, paraffin sections were cut one day before staining, dried over night at 37 °C, incubated for 1 hr at 60 °C and afterwards de-paraffinized/rehydrated in xylene and ascending RNA-pure EtOH series. After air drying, the slides were incubated for 20 min with RNAscope H_2O_2 at room temperature, washed in autoclaved double destilled H_2O and heated in RNAscope Target Retrieval Reagent for 10 min at 135 W and for 6 min for 180 W in a microwave. The slides were washed with autoclaved double destilled H_2O and 99 % RNA-pure EtOH and dried over night at room temperature in a wet chamber. Next, the sections were incubated for 30 min with RNAscope Protease Plus at 40 °C in a wet chamber followed by washing in autoclaved double destilled H_2O. Hybridization was conducted by applying two drops of the respective probe on the section for 2 hrs at 40 °C in a wet chamber. Afterwards the slides were washed for 4 min in 1 x RNAscope Wash Buffer and incubated with two drops of AMP1 (30 min), AMP2 (15 min), AMP3 (30 min), AMP4 (15 min), AMP5 (1 hr) and AMP6 (15 min), respectively at 40 °C in a wet chamber, including respective washing steps between the AMP probes each for 4 min in 1 x RNAscope Wash Buffer. After AMP6-incubation the slides were washed two times with 1 x

RNAscope Wash Buffer for 4 min. Signal detection was performed with the Fast-RED reagent for 10 min at room temperature in a wet chamber. Subsequent antibody staining was performed directly after the signal detection with Fast RED. For combined RNAScope/immunofluorescent stainings RNAScope staining was conducted as described above followed incubation with primary and secondary antibodies for immunofluorescent stainings. For quantification of *Gli1*$^+$ and *Gli2*$^+$ somatotrophs or FSC, all Gh$^+$, *Gli1*$^+$ Gh$^+$ and *Gli2*$^+$ Gh$^+$ cells out of 182-300 nucleated cells/pituitary of 4 wildtype C57BL/6N pituitary glands and all EGFP$^+$, *Gli1*$^+$ EGFP$^+$ and *Gli2*$^+$ EGFP$^+$ out of 123-241 nucleated cells/pituitary of 5 or 4 *S100b-EGFP* pituitary glands, respectively, were counted at 1,800-fold magnification.

Fluorescent or immunohistological stainings were examined with an Olympus confocal laser scanning microscope equipped with Fluoview FV100 software or an Olympus BX60 equipped with CellSense software (Olympus corporation, Shinjuku, Japan), respectively.

Transcriptome analyses

Three biological replicates of RNA from TtT/GF cells treated with either 100 nM SAG or solvent (see above) were analysed. RNA quality control (Fragment Analyzer, Agilent Technologies, USA), cDNA library preparation (TruSeq® RNA Sample Preparation v2; Illumina, San Diego, USA) and RNA sequencing (HiSeq 4000; Illumina) were performed at the NGS Service Facility for Integrative Genomics, Institute of Human Genetics, University Medical Center Göttingen, Germany. In detail, all fastq files are single end reads with 51 basepair readlength. They were aligned against the reference of mus musculus (Mus_musculus.GRCh38 Ensembl 96) genome with the RNA-Seq splice aware aligner STAR (Dobin et al., 2013) version 2.7.0f. Afterwards, aligned reads were counted towards their features (genes) with rsem calculate

expression (Li and Dewey, 2011) (RSEM v1.1.1), not only utilizing unique matched reads, but multi matched reads as well, allowing for the most comprehensive usage of the input read data. The resulting expected counts were the baseline input for further analysis in the programming language R (3.6.1) using the limma + voom (Law et al., 2014) (limma version 3.40.6) package for differential gene expression analysis. Following GSEA 6tilizing the clusterProfiler v3.12 (Yu et al., 2012) package in R. For quality control during the analysis pipeline multiQC (Ewels et al., 2016) version 1.8, evaluating the output of fastqc (https://www.bioinformatics.babraham.ac.uk/projects/fastqc/) and the log files of STAR, was used. Final data visualization was performed with Rstudio (Rstudio, Inc., USA) and GraphPadPrism 6 (GraphPad Software, Inc., San Diego, USA).

Supplemental Figure legends

Figure S1: Long-term characterization of the specificity and inducibility of the *PomcCreERT2*-deleter in adult pituitary glands. (A) Experimental setup and **(B)** representative immunofluorescence analyses of adult *Pomc/tdT* pituitaries 7, 14, 50, 100, 150, 200 and 250 days post-tamoxifen. Analyses were conducted on pituitaries of at least 3 animals per cohort. AL: anterior lobe; IL: intermediate lobe. Arrows: double positive cells. Scale bars: 500 µm (left panels), 10 µm (right panels).

Figure S2: Characterization of the murine FSC cell line TtT/GF, the rat somatotroph cell line GH3 and the murine corticotroph cell line AtT-20. (A,B) TtT/GF cells grow with a stellate-shaped morphology (A left), express the FSC- and stem cell marker Sox2 (A right) and show high expression of the FSC marker genes *S100b*, *Vegfa*, *Mif* and *Fst* (B). **(C-E)** GH3 cells express Gh (C, left) and Ghrhr protein (C right, E) as well as high levels of *Gh* transcripts (D). **(F,G)** AtT-20 cells express Pomc (F left) and Acth (F right) protein as well as high levels of *Pomc* transcripts (G). Gene expression levels were normalized to *18S* rRNA expression and to the respective gene expression levels of NIH/3T3 cells (dotted lines in B and D). *Pomc* transcript levels of NIH/3T3, TtT/GF and GH3 remained below detection level. Each open circle indicates one biological replicate measured in technical triplicates. Mean +/- SEM. Significant differences were tested using the non-parametric Holm-Sidak method. Significant differences to the respective base line (dotted lines) are indicated by asterisks above the data. *, P=0.05; **, P=0.01; ***, P=0.001; ****, P=0.0001. glycosyl. Ghrhr: glycosylated Ghrhr variants (Chu et al., 2016). Scale bars: 200 µm (A left), 10 µm (A right, C, F)

Figure S3: Characterization of Hh signaling activity of the murine FSC cell line TtT/GF, the rat somatotroph cell line GH3 and the murine corticotroph cell line AtT-20. (A) *Gli1* expression analysis of TtT/GF, GH3 and AtT-20 cells compared to the fibroblast cell line NIH/3T3. Gene expression levels were

normalized to *18S* rRNA expression and to the respective gene expression levels of NIH/3T3 cells (dotted line). Each open circle indicates one biological replicate measured in technical triplicates. Mean +/- SEM. Significant differences were tested using the non-parametric Holm-Sidak method. Significant differences to the respective base line (dotted lines) are indicated by asterisks above the data. *, *P*=0.05; ***, *P*=0.001; ****, *P*=0.0001. (B-D). Representative double immunofluorescent stainings of TtT/GF (B), GH3 (C) and AtT-20 cells (D) for analysis of the Smo location within primary cilia. Scale bars: 33 µm (B), 5 µm (C, D).

Figure S4: Smoothened Agonist treatment of the murine FSC cell line TtT/GF, the rat somatotroph cell line GH3 and the murine corticotroph cell line AtT-20. (A-C) Gene expression analyses of TtT/GF (A), GH3 (B) and AtT-20 (C) cells after serum starvation followed by 48 h 100 nM Smoothened Agonist or solvent treatment (dotted lines) dissolved in the respective starvation conditions. Gene expression levels were normalized to *18S* rRNA expression and to the respective gene expression levels of solvent-treated control cells (dotted line). Each open circle indicates one biological replicate measured in technical triplicates. Mean +/- SEM. Significant differences were tested using the non-parametric Holm-Sidak method. Significant differences to the respective base line (dotted lines) are indicated by asterisks above the data. *, *P*=0.05; **, *P*=0.01; ****, *P*=0.0001.

Figure S5: Medium of Smoothened Agonist-stimulated TtT/GF cells does not impact on Hh signaling activity or *Pomc* expression levels of AtT-20 cells. Gene expression analyses of Hh signaling target genes (A,B) and *Pomc* (B) of **(A)** TtT/GF cells after serum starvation followed by 48 h 100 nM Smoothened Agonist (SAG) or solvent treatment (dotted line) in AtT-20 serum starvation medium and **(B)** of AtT-20 cells after serum starvation followed by 48 h 100 nM SAG (gray circles, same data as shown in Fig. S5C) or solvent treatment (dotted line) or by 48 h incubation with conditioned media from TtT/GF cells (shown in A) treated with SAG (CM-TtT/GF_{SAG}, red circles) or solvent (dotted line). **(C)** Acth protein concentration in supernatant

of AtT-20 cells after serum starvation followed by 48 h incubation with conditioned media from TtT/GF cells (shown in A) treated with SAG (CM-TtT/GF_{SAG}, red circles) or solvent (dotted line). Gene expression levels were normalized to *18S* rRNA expression and to the respective gene expression levels of solvent-treated control cells (dotted line). Acth concentration was normalized to the Acth concentration of solvent-treated control cells (dotted lines). Each open circle indicates one biological replicate measured in technical triplicates. Mean +/- SEM. Significant differences were tested using the non-parametric Holm-Sidak method. Significant differences to the respective base line (dotted lines) are indicated by asterisks above the data. *, *P*=0.05; **, *P*=0.01; ****, *P*=0.0001.

Figure S6: Graphical representation of gene expression levels of the murine FSC cell line TtT/GF after Smoothened Agonist treatment determined by comparative transcriptome analyses. Expression profile of **(A)** differential expressed and **(B)** FSC marker genes of Smoothened Agonist (SAG)- versus solvent-treated (DMSO) TtT/GF cells based on transcriptome analyses (see Fig. 7). Gene expression of SAG-treated cells were normalized to solvent-treated controls (dotted lines). Differential expression (cut off 0.8- or 1.2-fold change, grey lines) with adjusted *P* values (non-parametric Holm-Sidak method) below 0.05 were consider to be significant. *, *P*=0.05; **, *P*=0.01.

Figure S7: EIA-based Vip protein measurements. (A) Standard curve of the EIA-based measurement of Vip protein concentrations and **(B)** absolute and **(C)** relative Vip protein levels in the supernatant of SAG- (CM-TtT/GF_{SAG}) or solvent-treated (CoM-TtT/GF) TtT/GF cells of 3 biological independent experiments each conducted in technical duplicates (biological replicate #1) or in triplicates (biological replicates #2 and #3). For calculation of relative Vip protein levels (C) Vip concentrations of CoM-TtT/GF of the respective biological replicates were set to 1. Grey dotted vertical lines indicate the range of Vip concentrations in the tested samples.

Supplemental References

Becker, M., Bauer, J., Pyczek, J., Konig, S., Mullen, A., Rabe, H., Schon, M. P., Uhmann, A. & Hahn, H. 2020. WIF1 Suppresses the Generation of Suprabasal Cells in Acanthotic Skin and Growth of Basal Cell Carcinomas upon Forced Overexpression. *J Invest Dermatol*.

Chu, W. K., Law, K. S., Chan, S. O., Yam, J. C., Chen, L. J., Zhang, H., Cheung, H. S., Block, N. L., Schally, A. V. & Pang, C. P. 2016. Antagonists of growth hormone-releasing hormone receptor induce apoptosis specifically in retinoblastoma cells. *Proc Natl Acad Sci U S A*, 113, 14396-14401.

Dobin, A., Davis, C. A., Schlesinger, F., Drenkow, J., Zaleski, C., Jha, S., Batut, P., Chaisson, M. & Gingeras, T. R. 2013. STAR: ultrafast universal RNA-seq aligner. *Bioinformatics*, 29, 15-21.

Ewels, P., Magnusson, M., Lundin, S. & Kaller, M. 2016. MultiQC: summarize analysis results for multiple tools and samples in a single report. *Bioinformatics*, 32, 3047-8.

Law, C. W., Chen, Y., Shi, W. & Smyth, G. K. 2014. voom: Precision weights unlock linear model analysis tools for RNA-seq read counts. *Genome Biol*, 15, R29.

Li, B. & Dewey, C. N. 2011. RSEM: accurate transcript quantification from RNA-Seq data with or without a reference genome. *BMC Bioinformatics*, 12, 323.

Pyczek, J., Buslei, R., Schult, D., Holsken, A., Buchfelder, M., Hess, I., Hahn, H. & Uhmann, A. 2016. Hedgehog signaling activation induces stem cell proliferation and hormone release in the adult pituitary gland. *Sci Rep*, 6, 24928.

Yu, G., Wang, L. G., Han, Y. & He, Q. Y. 2012. clusterProfiler: an R package for comparing biological themes among gene clusters. *OMICS*, 16, 284-7.
