# Supplementary figures and images for: Hedgehog signaling in endocrine and folliculo-stellate cells of the adult pituitary
Source: J Endocrinol. 2021 Jan 15;248(3):303–16. doi: 10.1530/JOE-20-0388 (PMC7983331; doi:10.1530/JOE-20-0388)

A

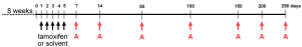

B

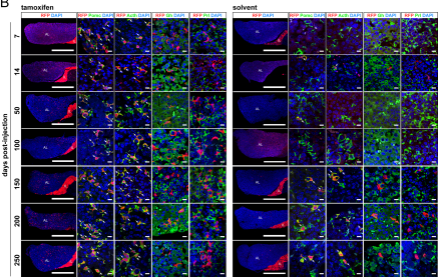

Supplement: Figure S1: Long-term characterization of the specificity and inducibility of the PomcCreERT2-deleter in adult pituitary glands. (A) Experimental setup and (B) representative immunofluorescence analyses of adult Pomc/tdT pituitaries 7, 14, 50, 100, 150, 200 and 250 days post-tamoxifen. Analyses were  [file supplementary_figure_1.pdf]

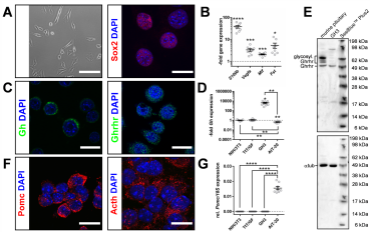

Supplement: Figure S2: Characterization of the murine FSC cell line TtT/GF, the rat somatotroph cell line GH3 and the murine corticotroph cell line AtT-20. (A,B) TtT/GF cells grow with a stellate-shaped morphology (A left), express the FSC- and stem cell marker Sox2 (A right) and show high expression of the FSC [file supplementary_figure_2.pdf]

**A**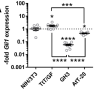**B**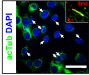**C**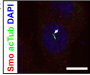**D**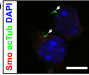

Supplement: Figure S3: Characterization of Hh signaling activity of the murine FSC cell line TtT/GF, the rat somatotroph cell line GH3 and the murine corticotroph cell line AtT-20. (A) Gli1 expression analysis of TtT/GF, GH3 and AtT-20 cells compared to the fibroblast cell line NIH/3T3. Gene expression levels w [file supplementary_figure_3.pdf]

**A**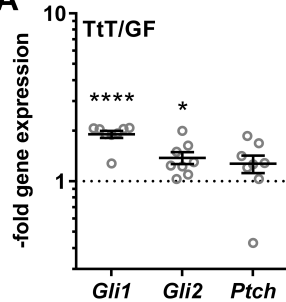**B**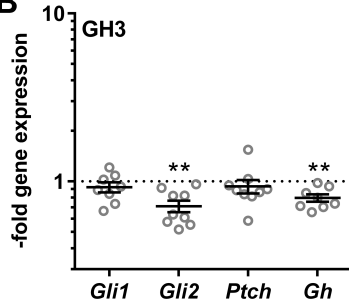**C**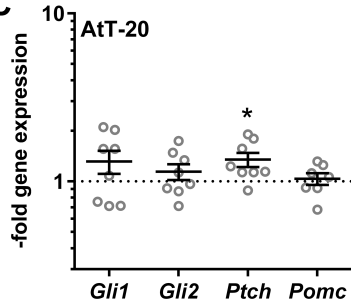

Supplement: Figure S4: Smoothened Agonist treatment of the murine FSC cell line TtT/GF, the rat somatotroph cell line GH3 and the murine corticotroph cell line AtT-20. (A-C) Gene expression analyses of TtT/GF (A), GH3 (B) and AtT-20 (C) cells after serum starvation followed by 48 h 100 nM Smoothened Agonist or  [file supplementary_figure_4.pdf]

**A**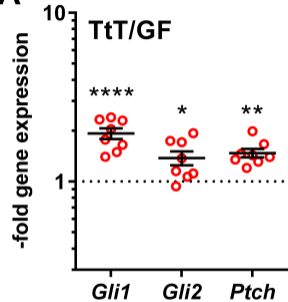**B**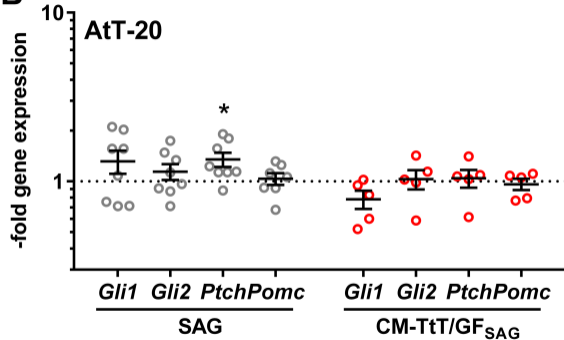**C**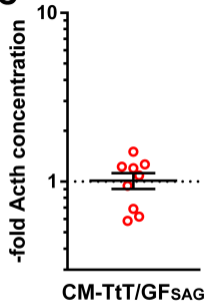

Supplement: Figure S5: Medium of Smoothened Agonist-stimulated TtT/GF cells does not impact on Hh signaling activity or Pomc expression levels of AtT-20 cells. Gene expression analyses of Hh signaling target genes (A,B) and Pomc (B) of (A) TtT/GF cells after serum starvation followed by 48 h 100 nM Smoothened A [file supplementary_figure_5.pdf]

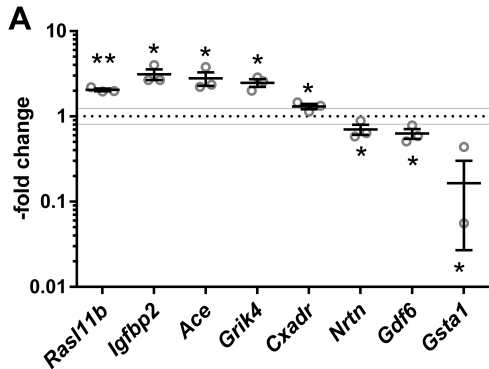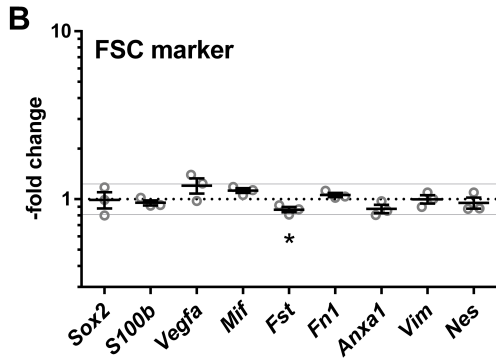

Supplement: Figure S6: Graphical representation of gene expression levels of the murine FSC cell line TtT/GF after Smoothened Agonist treatment determined by comparative transcriptome analyses. Expression profile of (A) differential expressed and (B) FSC marker genes of Smoothened Agonist (SAG)- versus solvent- [file supplementary_figure_6.pdf]

**A**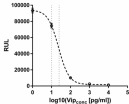**B**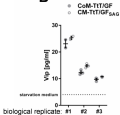**C**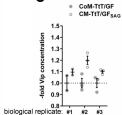

Supplement: Figure S7: EIA-based Vip protein measurements. (A) Standard curve of the EIA-based measurement of Vip protein concentrations and (B) absolute and (C) relative Vip protein levels in the supernatant of SAG- (CM-TtT/GFSAG) or solvent-treated (CoM-TtT/GF) TtT/GF cells of 3 biological independent experim [file supplementary_figure_7.pdf]
